# Identification of potential drug targets for rheumatoid arthritis from genetic insights: a Mendelian randomization study

**DOI:** 10.1186/s12967-023-04474-z

**Published:** 2023-09-11

**Authors:** Yu Cao, Ying Yang, Qingfeng Hu, Guojun Wei

**Affiliations:** 1 Department of Orthopedics, The Fourth Affiliated Hospital, School of Medicine, Zhejiang University, No. N1, Shangcheng Avenue, Yiwu City, Zhejiang Province China; 2 Xiang’an Hospital of Xiamen University, Xiamen, China

**Keywords:** Rheumatoid arthritis, Mendelian randomization, Drug targets, Genetics

## Abstract

**Introduction:**

Rheumatoid arthritis (RA) is a chronic inflammatory illness that mostly affects the joints of the hands and feet and can reduce life expectancy by an average of 3 to 10 years. Although tremendous progress has been achieved in the treatment of RA, a large minority of patients continue to respond poorly to existing medications, owing in part to a lack of appropriate therapeutic targets.

**Methods:**

To find therapeutic targets for RA, a Mendelian randomization (MR) was performed. Cis-expression quantitative trait loci (cis-eQTL, exposure) data were obtained from the eQTLGen Consortium (sample size 31,684). Summary statistics for RA (outcome) were obtained from two largest independent cohorts: sample sizes of 97,173 (22,350 cases and 74,823 controls) and 269,377 (8279 cases and 261,098), respectively. Colocalisation analysis was used to test whether RA risk and gene expression were driven by common SNPs. Drug prediction and molecular docking was further used to validate the medicinal value of drug targets.

**Results:**

Seven drug targets were significant in both cohorts in MR analysis and supported by localization. PheWAS at the gene level showed only ATP2A1 associated with other traits. These genes are strongly associated with immune function in terms of biological significance. Molecular docking showed excellent binding for drugs and proteins with available structural data.

**Conclusion:**

This study identifies seven potential drug targets for RA. Drugs designed to target these genes have a higher chance of success in clinical trials and is expected to help prioritise RA drug development and save on drug development costs.

**Supplementary Information:**

The online version contains supplementary material available at 10.1186/s12967-023-04474-z.

## Introduction

Rheumatoid arthritis (RA) is a chronic inflammatory immune-mediated disease characterized by synovitis and cartilage destruction, primarily affecting the synovial membranes, tendon sheaths, and synovial bursae of the joints [1], and manifesting as joint pain, stiffness, swelling, deformity, and functional impairment [2]. It has a roughly 1% worldwide prevalence and is the 42nd most common debilitating disease in the world [3]. Its prevalence is rising as the world's population ages. Because of the high mortality and morbidity of RA, patients have a terrible quality of life and a huge economic burden on society. The National Audit Office estimates that RA costs the UK roughly £560 million per year in healthcare expenditures, not counting the cost of sickness absence and work-related impairment [4].

There is no cure for rheumatoid arthritis, so patients need to take long-term medication to reduce their signs and symptoms. These medications are often classified as disease-modifying antirheumatic drugs, glucocorticoids, nonsteroidal anti-inflammatory medicines and biologic drugs [5]. At the same time, the treatment of RA has improved considerably thanks to new targeted therapies that interfere with specific pathways of inflammation and immune response, such as antibodies against inflammatory cytokines or antibodies against immune cell surface molecules [6]. Nonetheless, a considerable minority of RA patients continue to fail to react to existing therapies [7]. In other words, despite the significant improvements in the treatment of RA with the introduction of biological and targeted synthetic disease-modifying anti-rheumatic drugs, a significant proportion of patients remain asymptomatic. These patients can be considered as 'Difficult-to-Treat' RA patients, and this group of patients represents a huge treatment challenge for both the healthcare team and the patient. In addition to new management strategies, optimal care for these patients requires new therapeutic drug targets [6].

Incorporating genetics into medication development might be one of the most effective strategies to enhance this process, as genetically backed therapies are far more likely to succeed in clinical trials [8–10]. Proteins encoded by druggable genes have become targets for drugs, or potentially for small molecules or monoclonal antibodies [11, 12]. Although genome-wide association studies (GWAS) have been efficient in identifying SNPs (Single Nucleotide Polymorphisms) linked with RA risk [13–15], GWAS methods cannot consistently identify causative genes and directly drive medication development.

Mendelian randomization (MR) is a statistical analysis of genetics that can be used to predict drug efficacy by mimicking randomised controlled trials [16–19]. SNPs (Expression Quantitative Trait Loci, eQTLs) linked to changes in gene expression may be comparable to long-term exposure to medications that target the encoded proteins [11, 20]. The GWAS for outcome (RA) may then be used to derive association data between the same genetic variations (SNPs) and disease (RA). Using MR, it is possible to integrate data on SNP-gene expression and SNP-disease connection to establish a causal relationship between exposure and outcome. A strong MR research may be designed using publically accessible data from two large-scale GWAS because exposure and outcome can be evaluated in two independent cohorts. Furthermore, people are randomly allocated variations with either high or low expression levels of druggable genes after fertilization, and individuals are often ignorant of their genotype, therefore MR investigations are akin to blinded trials [21]. However, it is important to acknowledge the limitations of the Mendelian randomization (MR) approach used in this study. One potential limitation is the presence of horizontal pleiotropy, where the instrumental variables may have direct effects on outcomes other than the exposure of interest. This can introduce bias in the estimation of causal effects. Violations of instrumental variable assumptions can also affect the validity of MR analyses. It is crucial to carefully consider and address these limitations and potential sources of bias in the interpretation of the results.

In this study, we propose several innovative findings in the field of rheumatoid arthritis (RA) research. Firstly, we identify new therapeutic targets for RA using the Mendelian randomization approach and large-scale genome-wide association study data. This approach combines cis-eQTL and RA risk association data to establish a causal relationship between exposure and outcome, improving drug efficacy prediction. Secondly, we validate the pharmacological activity of seven potential RA drug targets through drug prediction and molecular docking studies. These targets, closely related to immune function, are assessed for feasibility and potential drug candidates by evaluating their binding affinity and interaction patterns with drugs.

Additionally, we conduct gene co-localization analysis to confirm the shared driving factors between potential therapeutic targets and RA risk. This analysis helps determine the causal relationship between treatment targets and the disease, excluding potential confounding factors. Furthermore, our PheWAS analysis explores the associations between potential therapeutic targets and other characteristics, providing valuable insights into their multifunctionality and potential impact mechanisms for further research and the development of related treatment strategies. Lastly, gene enrichment analysis and protein–protein interaction network construction reveal the functional characteristics and biological relevance of potential therapeutic targets, deepening our understanding of their mechanisms in RA development and treatment.

In summary, our study offers important insights into the discovery of new therapeutic targets for RA. By integrating Mendelian randomization, drug prediction, gene co-localization analysis, PheWAS, gene enrichment analysis, and protein–protein interaction network construction, we provide valuable guidance for the development of more effective and targeted treatment approaches.

## Materials and Methods

### Exposure data

eQTLs data were obtained from eQTLGen Consortium (https://eqtlgen.org/). In brief, the eQTLGen data set contained 16,987 genes and 31,684 cis-eQTLs in blood samples from mostly healthy European individuals. A full description of the data can be found in the original article [22]. On March 13, 2023, the entire cis-eQTLs data and allele frequency statistics were acquired from the eQTLGen consortium. The list of druggable genes is from a previous study. It was designed as a computational method and combined with data from many existing genome-wide association studies to identify druggable proteins, linking them to known drugs, to propose 4463 druggable genes [12]. Considering that eQTLs are closer to the gene of interest in drug development studies and have more direct regulation of gene expression, the eQTLs used in this study were limited to SNPs with 5 kb upstream of the starting point or 5 kb downstream of the endpoint of a druggable gene. eQTLs for 2554 druggable genes were finally obtained.

### Outcome data

GWAS data for the RA discovery Cohort were obtained from a previous large multi-ethnic study [14]. This study included 35,871 RA cases and 24,0149 controls from 37 cohorts with European, East Asian, African, South Asian and Arab ancestry. All RA cases satisfied the 1987 American College of Rheumatology (ACR) criteria [23] or the 2010 ACR/European League Against Rheumatism criteria [24], or were diagnosed with RA by a professional rheumatologist. 31,963 of the 35,871 cases had a known seropositive status; of these, 27,448 were seropositive and 4,515 were seronegative (rheumatoid factor or anti-citrullinated peptide antibodies were used to determine seropositivity). Detailed information on the data can be found in the original literature [14]. For consistency with the exposure data, GWAS data from the European pedigree sample of 22,350 cases and 74,823 controls were selected as outcome data for this study. GWAS data for the RA replication cohort containing 8279 cases and 261,098 controls were obtained from FinnGen Release 8 (https://www.finngen.fi/en) [25], which were released in December 2022.

### Mendelian randomisation analysis

The R package TwoSampleMR (version 0.5.6) was used to conclude the MR analysis [26]. Exposure and outcome data were imported and harmonised using the R package built-in function (harmonise_data). The genetic instrumental variables used for MR analysis were subject to three MR assumptions: (1) SNPs were directly linked with exposure (i.e. highly associated with at least one gene expression, FDR < 0.05); (2) SNPs were not associated with exposure-outcome confounders; and (3) SNPs affected outcome through exposure only [27]. Therefore, several quality controls were performed in this study. First, the exposure and outcome groups were nearly entirely composed of people of European ancestry, reducing any potential bias from population stratification. Second, to decrease bias caused by weak instrumental factors, instrumental variables having a F statistic of less than 10 [F = (beta/se)2] were deleted [28]. When there was genetic linkage between SNPs, a reference panel from the 1000 Genome Project [29] was used to remove linked SNPs at r2 < 0.2 and a cropping range of 10,000 Kb, retaining the most significant SNPs (with the smallest p-value) and ensuring independence between SNPs. Finally, Steiger filtering approach was used to exclude genes with more SNP explained outcome (RA) variance than exposed variation.

In the main analysis, MR estimates were calculated for each SNP using the Wald ratio method, and for genes with multiple instrumental variables, SNP estimates were meta-analysed using inverse variance weighted (IVW), MR-Egger and weighted median methods. IVW assumes that all genetic instruments are valid, and its statistical power is the highest of all methods when the assumption is valid [30]. The weighted median technique allows some (50%) instrumental variables to be invalid by weighting the MR estimates produced by each SNP according to their magnitude and providing an overall MR estimate based on the median with bootstrapped standard errors [31]. MR Egger allows for the presence of horizontal pleiotropy, i.e. some SNPs may have an influence on the outcome via a different pathway than the exposure of interest, albeit at the expense of diminished statistical power. The MR Egger intercept test (MR-Egger intercept test) can be used to determine whether or not there is horizontal pleiotropy present [32]. When these three methods yield directionally consistent estimates it indicates that pleiotropy does not bias the IVW estimates. For genes containing more than two instrumental variables, MR-Egger intercept tests were performed to test for the presence of horizontal pleiotropy (judged at P < 0.05), and Cochran's Q was determined using both the IVW and MR-Egger techniques to test for heterogeneity amongst Wald ratios [33]. The Bonferroni correction was applied to determine the adjusted significance threshold for multiple testing, taking into account the false positives caused by multiple testing. In the discovery cohort, a P value below 1.96E−5 (P = 0.05/2554) was defined as significant. For significant genes, quality control was performed by checking that the three methods were consistent in the direction of estimated effect and that the MR-Egger test had no horizontal pleiotropy. Significant genes that passed quality control were repeatedly validated in the FinnGen cohort, and associations with a P value below 0.0017 (P = 0.05/29) were considered significant.

### Colocalisation analysis

For genes that were significant in both cohorts, colocalisation analysis of RA risk was performed using the R package coloc [34]. Analyses were performed using SNPs harmonised by TwoSampleMR package with default priori probabilities: p1 = 1E−4, p2 = 1E−4, p12 = 1E−5. P1, p2, and p12 are predefined probability that the SNP in the test area is substantially linked with gene expression, RA risk, or both. The posterior probabilities derived from the colocalization analysis correspond to one of five hypotheses: PPH0, SNPs are not associated with either trait; PPH1, SNPs are associated with gene expression but not with RA risk; PPH2, associated with RA risk but not with gene expression; PPH3, associated with RA risk and gene expression but driven by different SNPs; PPH4, associated with RA risk and gene expression, was driven by common SNPs. The threshold of significance for colocalisation was set at PPH4 > 0.80, and genes that colocalised with RA could be considered as potential drug target genes.

### Phenome-wide association analysis

In order to further evaluate the horizontal pleiotropy of potential drug targets and possible side effects, a phenome-wide association study (PheWAS) was performed on AstraZeneca PheWAS Portal (https://azphewas.com/) [35]. The original study used data of ~ 15.5 K binary and ~ 1.5 K continuous phenotypes from a subset of approximately 450,000 exome sequencing participants published by UK Biobank. The full construction methodology can be found in the original article [35]. We performed multiple corrections and set a threshold of 2E−9 (as the default in the AstraZeneca PheWAS Portal) to account for the potential for false positives.

### Enrichment analysis

To investigate the functional characteristics and biological relevance of the identified prospective therapeutic target genes, Gene Ontology (GO) and Kyoto Encyclopedia of Genes and Genomes (KEGG) enrichment study were done using the R package clusterProfiler [36], Pathview [37]. GO includes three terms: biological process (BP), molecular function (MF), and cellular component (CC). The KEGG pathway can provide metabolic pathway information.

### Protein interaction network construction

By evaluating and analyzing protein–protein interaction (PPI) networks, one can gain a better knowledge of how one protein interacts with another intracellularly. In this study, the PPI network was built using STRING with a confidence score of 0.4 as the minimum needed interaction score and all other parameters left at default levels [38]. PPI results were further visualised by Cytoscape (V3.9.1) [39]. In addition, GeneMANIA (https://genemania.org/) was also used for PPI analysis [40].

### Candidate drug prediction

Assessing protein-drug interactions is important to understand whether target genes can be used as actual drug targets. This study will use the Drug Signatures Database (DSigDB, http://dsigdb.tanlab.org/DSigDBv1.0/) [41] to accomplish this. Specifically, with 22,527 gene sets and 17,389 distinct compounds spanning 19,531 genes, DSigDB is a sizable database that connects medicines and other chemicals to their target genes. The identified target genes are uploaded to DSigDB and drug candidates can be predicted to assess the medicinal activity of the target genes.

### Molecular docking

**Fig. 1:**
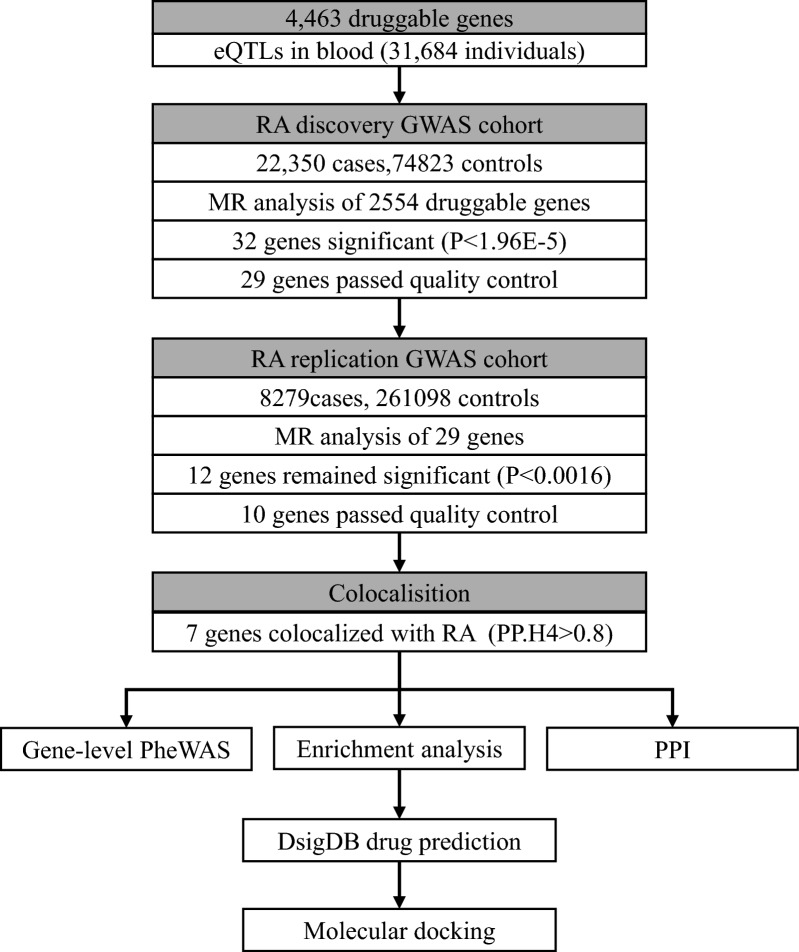
Overview of the study design

To further understand the effect of drug candidates on drug target genes and the druggability of target genes, this study further performed molecular docking at the atomic level to assess the binding energy and interaction pattern between drug candidates and their targets. Molecular docking simulations allow us to analyze the binding affinity and mode of interaction between ligands and drug targets. By identifying ligands with high binding affinity and favorable interaction patterns, we can prioritize drug targets for further experimental validation and optimize the design of potential drug candidates.In this study, AutodockVina 1.2.2 (http://autodock.scripps.edu/), a computerised protein–ligand docking software, was used to perform molecular docking of the top 5 significant drugs and the proteins encoded by the corresponding target genes [42]. Drug structure data were obtained from the PubChem Compound Database [43] (https://pubchem.ncbi.nlm.nih.gov/) and the corresponding IDs have been displayed in Table 4. Protein structure data were downloaded from the PDB (Protein Data Bank, http://www.rcsb.org/) and the corresponding PDB IDs can be viewed in Table 4 (except for ATP2A1 protein from EMBL-EBI (https://www.ebi.ac.uk/) Alphafold-based structure data). The final structures were obtained for five proteins and four drugs. To begin, all water molecules were removed from the protein and ligand files and polar hydrogen atoms were added. The grid boxes were centered to encompass each protein's structural domains and to allow for unrestricted molecular mobility. A grid point distance of 0.05 nm and a pocket size of 30 Å × 30 Å × 30 Å were used to build up the docking pocket. The entire molecular docking process was visualised in the model by Autodock Vina 1.2.2. Flow chart of this study was presented in Fig. 1.

## Results

### Thirty-two genes causally significantly associated with RA risk during the discovery phase

**Fig. 2:**
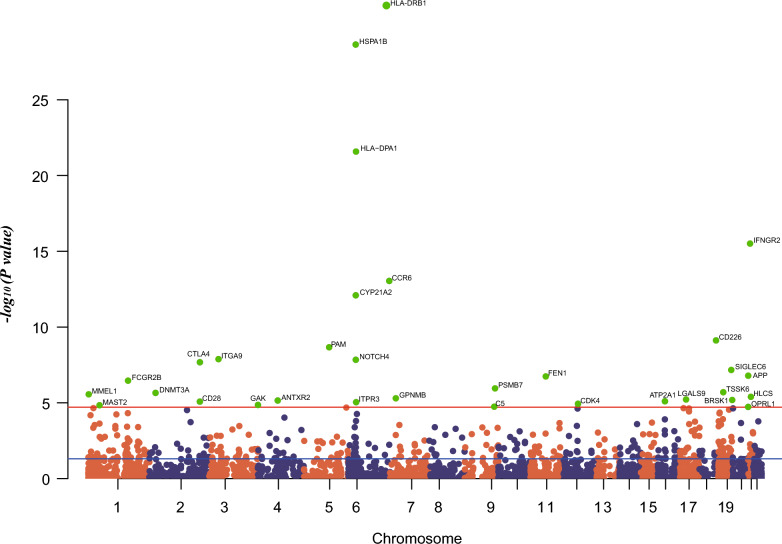
Manhattan plot of MR analysis in discovery phase. Significant genes were labelled and highlighted in green. The blue line represents the nominal significant threshold of 0.05. The red line represents the Bonferroni correction threshold of 1.96E−5

**Fig. 3:**
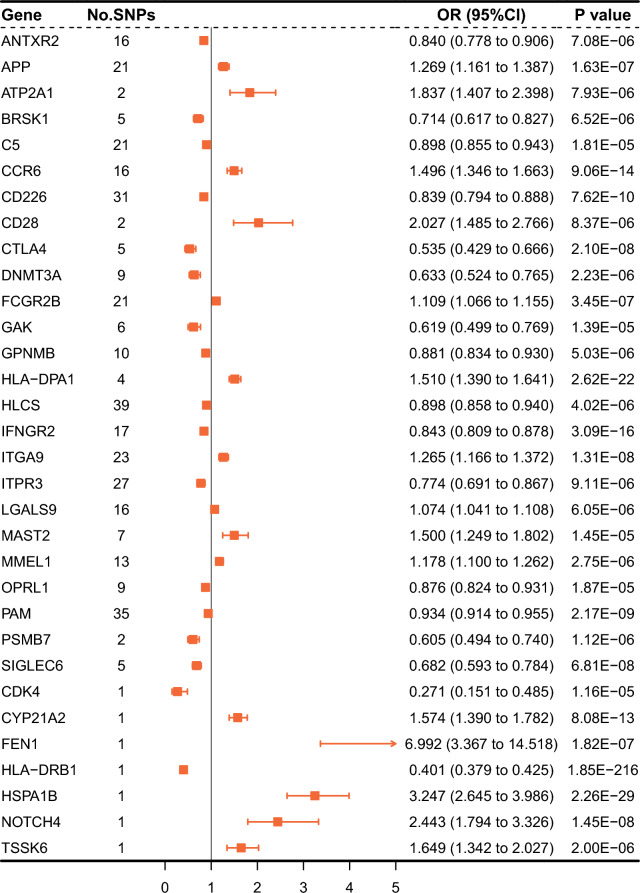
Forest plots displaying the findings from the discovery phase for 32 significant genes

The largest current GWAS for RA traits was a large multi-ethnic study in 2022, containing 22,350 cases and 74,823 controls of European ancestry [14]. As shown in Figs. 2, 3, in the discovery cohort, the expression of 32 genes was causally related with RA risk (p < 1.96E−5 = 0.05/2554, Bonferroni correction for 2554 drug targets). However, when sensitivity analyses were performed, the BRSK1 gene had inconsistent direction of effect values across the three methods (Additional file 1: Table S1), and the CD226 and MMEL1 genes failed the horizontal pleiotropy test (P < 0.05, Additional file 1: Table S2), so these three genes were excluded from subsequent analyses. The results of the heterogeneity test (Additional file 1: Table S3) indicated heterogeneity between the SNPs for ITPR3, HLA-DPA1, C5, MMEL1 and CTLA4.

### Replication phase 12 genes remain significant in an independent RA cohorts

**Fig. 4:**
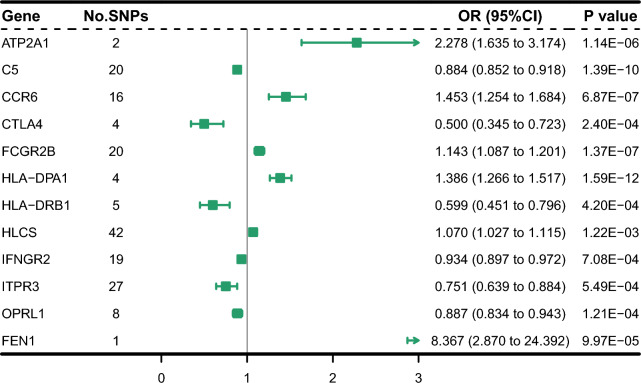
Forest plots displaying the findings from the replication phase for 12 significant genes

In the replication phase this study used GWAS data from the Finnish FinnGen database containing 8279 cases and 261,098 controls of European ancestry. The MR analysis was performed in the same way as in the discovery cohort. Using the Wald ratio or IVW approach, the genetic predicted expression of 12 genes was replicated as causally connected to RA risk, as shown in Fig. 4 (significant after Bonferroni correction at P < 0.0017 = 0.05/29). The CTLA4 gene was excluded from subsequent analyses due to inconsistent direction of effect values across the three methods (Additional file 1: Table S4). The horizontal pleiotropy test showed no significant pleiotropy for any of the genes (Additional file 1: Table S5). In the test for heterogeneity (Additional file 1: Table S6), the HLA-DRB1, ITPR3, CTLA4, FCGR2B, CCR6 and HLA-DPA1 genes showed inter-SNP heterogeneity. In addition, the HLCS gene was excluded from subsequent analyses due to inconsistency in the direction of effect estimates between the discovery and replication phase.

### Colocalization analysis

Previous studies have suggested that significant MR outcomes may arise from a locus in which SNPs are in close linkage disequilibrium and in which the SNP-exposure and SNP-outcome associations arise from two different causal SNPs, and therefore may lead to inferred false positive results [44]. Colocalisation analysis can be used to explore whether exposure and outcome share the same causal SNP when SNPs are clearly associated with both exposure and outcome [34]. Evidence suggests that proteins that have undergone both MR and colocalisation tests have greater potential to become drug targets and are more likely to be approved [45]. Therefore, the decision was made in this study to use discovery phase data to perform colocalisation on the 11 genes proposed in the previous analysis. As shown in Table 1, seven of the 11 proteins (CCR6, HLA-DPA1, HLA-DRB1, IFNGR2, C5, ATP2A1 and FEN1) showed strong evidence of colocalisation with RA (PP.H4 > 0.8) and could be candidate drug target genes.

**Table 1:** Colocalization results of eQTLs for 11 genes with RA-associated SNPs

Gene	PP.H0	PP.H1	PP.H2	PP.H3	PP.H4
CCR6	0.000	0.000	0.000	0.000	1.000
HLA-DPA1	0.000	0.000	0.000	0.000	1.000
HLA-DRB1	0.000	0.000	0.000	0.000	1.000
IFNGR2	0.000	0.000	0.000	0.000	1.000
C5	0.000	0.000	0.000	0.000	1.000
ATP2A1	0.000	0.014	0.000	0.000	0.986
FEN1	0.002	0.000	0.013	0.000	0.984
OPRL1	0.000	0.215	0.000	0.000	0.785
FCGR2B	0.000	0.620	0.000	0.338	0.042
ITPR3	0.000	0.000	0.000	1.000	0.000

PP.H0–PP.H4 represent the posterior probabilities of different hypotheses

PP.H4 > 0.8 represents a strong colocalization between gene expression and RA risk

### PheWAS

To further assess whether the seven potential drug target genes identified would have beneficial or deleterious effects on other traits and whether there was potential pleiotropy that was not captured by the MR-Egger intercept test, this study used 17,361 dichotomous phenotypes and 1419 quantitative phenotypes from the AstraZeneca PheWAS Portal database [35] to perform PheWAS at the gene level. PheWAS results can be interpreted as the association of genetically determined protein expression with specific diseases or traits. As shown in Table 2 and Additional file 1: Figs. S1–S14, with the exception of ATP2A1, none of the six drug targets were significantly associated with other traits at the gene level (P < 5E−8 for genomic association), suggesting that the potential side effects of drugs acting on these targets and the presence of horizontal pleiotropy in these genes are likely to be small, further indicating the reliability of the results of this study. In contrast, ATP2A1 was positively associated with Hand Grip Strength and negatively associated with Impedance of Arm, suggesting that RA drugs acting on ATP2A1 gene may affect both traits, and that the MR analysis of the ATP2A1 gene may have a pleiotropic effect on the results.

**Table 2:** Traits Significantly associated with APT2A1 using AstraZeneca PheWAS portal

Phenotype	Collapsing model	P value	No. samples	Effect size
Hand grip strength (left)	Flexdmg	1.34E−11	393,020	0.07
Hand grip strength (right)	Flexdmg	8.11E−11	393,037	0.07
Hand grip strength (left)	Ptvraredmg	9.84E−11	393,020	0.08
Impedance of arm (left)	Ptvraredmg	2.24E−10	387,633	–0.08
Hand grip strength (right)	Ptvraredmg	2.97E−10	393,037	0.07
Impedance of arm (right)	ptvraredmg	7.99E−10	387,617	– 0.07

### Enrichment analysis

**Fig. 5:**
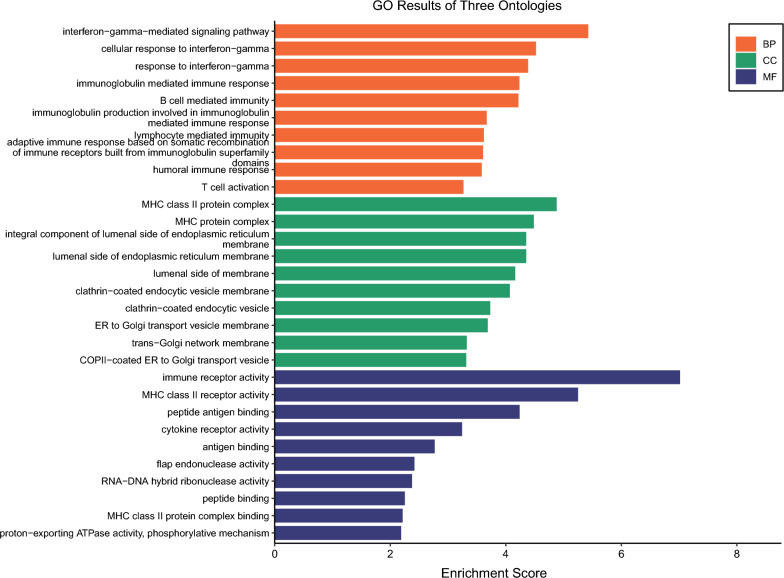
GO enrichment results for three terms

**Fig. 6:**
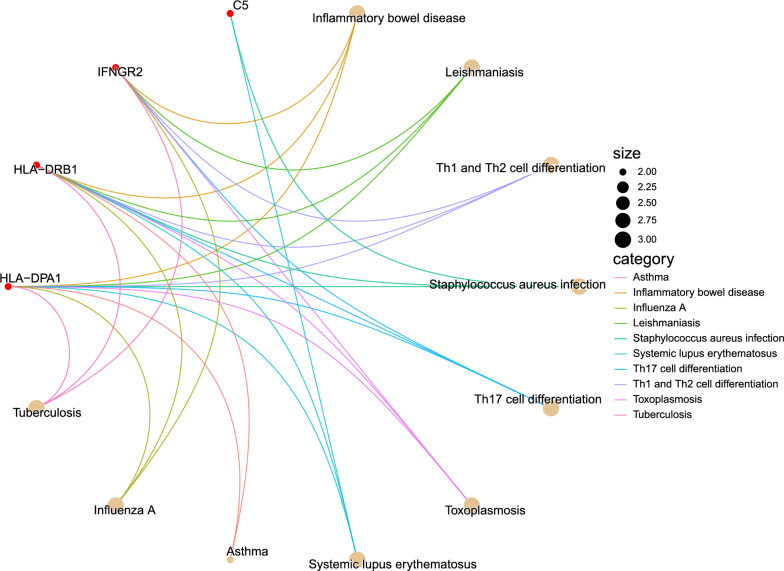
KEGG enrichment results

GO enrichment analysis is commonly used to show interactions between genes and terms, while KEGG enrichment analysis can illustrate the relationship between genes and functional pathways [46]. As shown in Fig. 5, the most significant pathways in the BP category were all associated with interferon-gamma (interferon-gamma-mediated signaling pathway, cellular response to interferon-gamma, response to interferon-gamma). In class CC, drug target genes are similarly enriched for immune and endoplasmic reticulum-related components (MHC class II protein complex, MHC protein complex, integral component of lumenal side of endoplasmic reticulum lumenal side of endoplasmic reticulum membrane and lumenal side of endoplasmic reticulum membrane), which is consistent with previous studies [47]. Furthermore, in terms of MF, these genes are also involved in functions strongly associated with immunity (immune receptor activity, MHC class II receptor activity, peptide antigen binding, cytokine receptor activity and antigen binding). As shown in Fig. 6**,** the first three pathways analysed by KEGG enrichment are Inflammatory bowel disease (IBD), Leishmaniasis, Th1 and Th2 cell differentiation, of which IBD is an autoimmune disease. IBD is an autoimmune disease and Leishmaniasis is a disease caused by a parasitic infection, all three of which are closely linked to the immune response.

### PPI networks

**Fig. 7:**
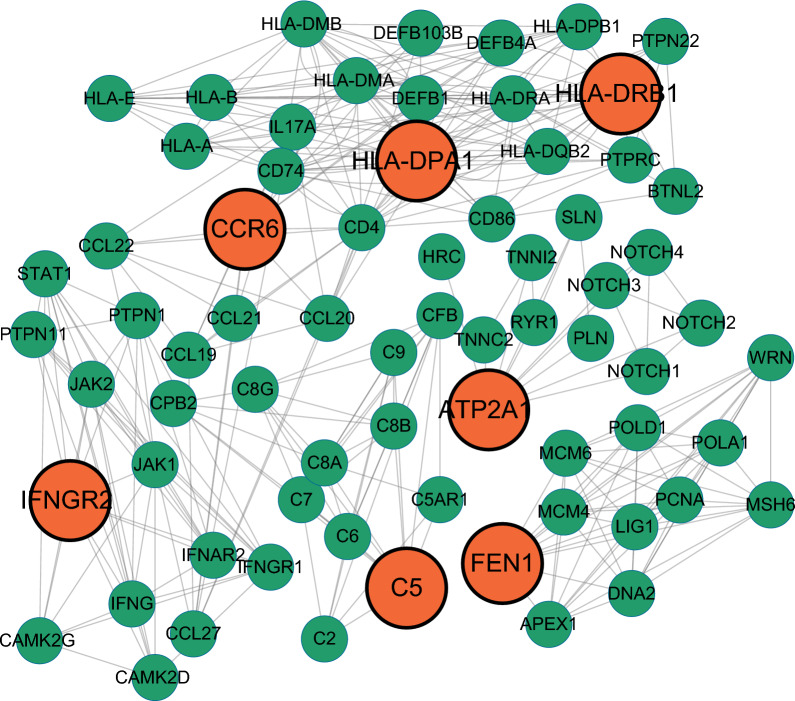
PPI network built with STRING

The seven drug target genes were loaded into the STRING (https://cn.string-db.org/) database for network creation, and the resultant files were imported into Cytoscape for visualization. Figure 7 depicts the interactions of the seven drug targets with other proteins in a 50-node, 375-edge PPI network.

**Fig. 8:**
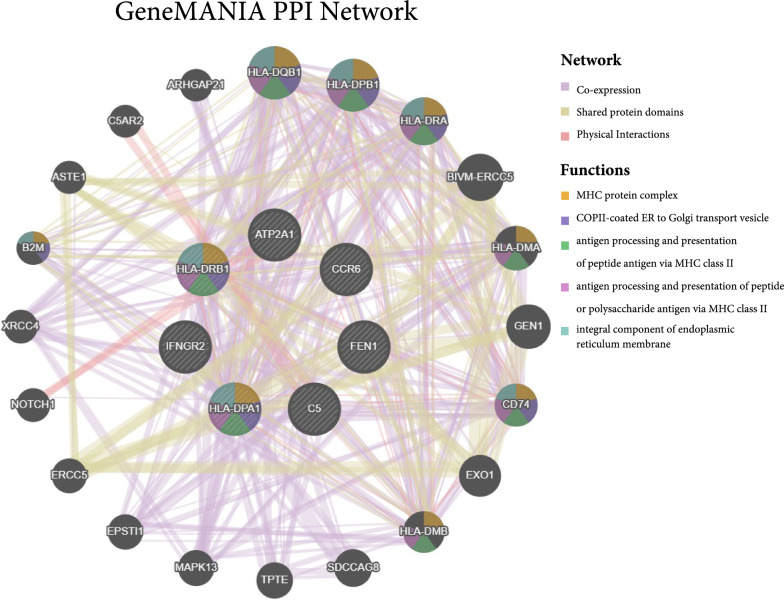
PPI network built with GeneMANIA. Each circle is coloured to indicate the functional pathway in which each gene is involved

For the PPI network constructed using GeneMANIA (https://genemania.org/), in addition to the 7 drug targets, the network includes an additional 20 potentially interacting genes for a total of 490 interaction links (Fig. 8). These linkages included co-expression (84.47%), common protein structural domains (9.90%) and physical interactions (5.63%). The functional analysis of the network depicts the role of drug targets and associated genes and their functions. Network functional analysis showed similar results to the previous enrichment analysis, all showing a strong immune functional correlation, consistent with the autoimmune disease nature of RA.

### Candidate drug prediction

The DSigDB database was used in this study to make predictions of potentially effective intervention drugs. Based on adjusted p-values, the top 10 potential chemical compounds were shown (Table 3). The results showed that rhubarb acid (Rhein TTD 00010611/ CTD 00001002) and tyrosine phosphorylation inhibitor (Tyrphostin AG 538 TTD 00011608) were the two most significant drugs, linked to FEN1 and CCR6. In contrast, quercetin (quercetin CTD 00006679) and arsenic (ARSENIC CTD 00005442) interacted with the most genes.

**Table 3:** Candidate drug predicted using DSigDB

Drug names	P-value	Adjusted P-value	Genes
Rhein TTD 00010611	0.000	0.014	FEN1; CCR6
Rhein CTD 00001002	0.000	0.014	FEN1; CCR6
Tyrphostin AG 538 TTD 00011608	0.000	0.014	FEN1; CCR6
TITANIUM DIOXIDE CTD 00000489	0.001	0.045	C5; IFNGR2
quercetin CTD 00006679	0.002	0.045	C5; FEN1; IFNGR2; ATP2A1;CCR6
(-)-isoprenaline HL60 UP	0.002	0.045	HLA-DRB1; HLA-DPA1
ARSENIC CTD 00005442	0.002	0.045	CCR6; HLA-DRB1; HLA-DPA1
MALEIMIDE CTD 00001979	0.004	0.045	CCR6
p-benzoquinone TTD 00009995	0.004	0.045	CCR6
sphingosine CTD 00006772	0.004	0.045	CCR6

### Molecular docking

**Fig. 9:**
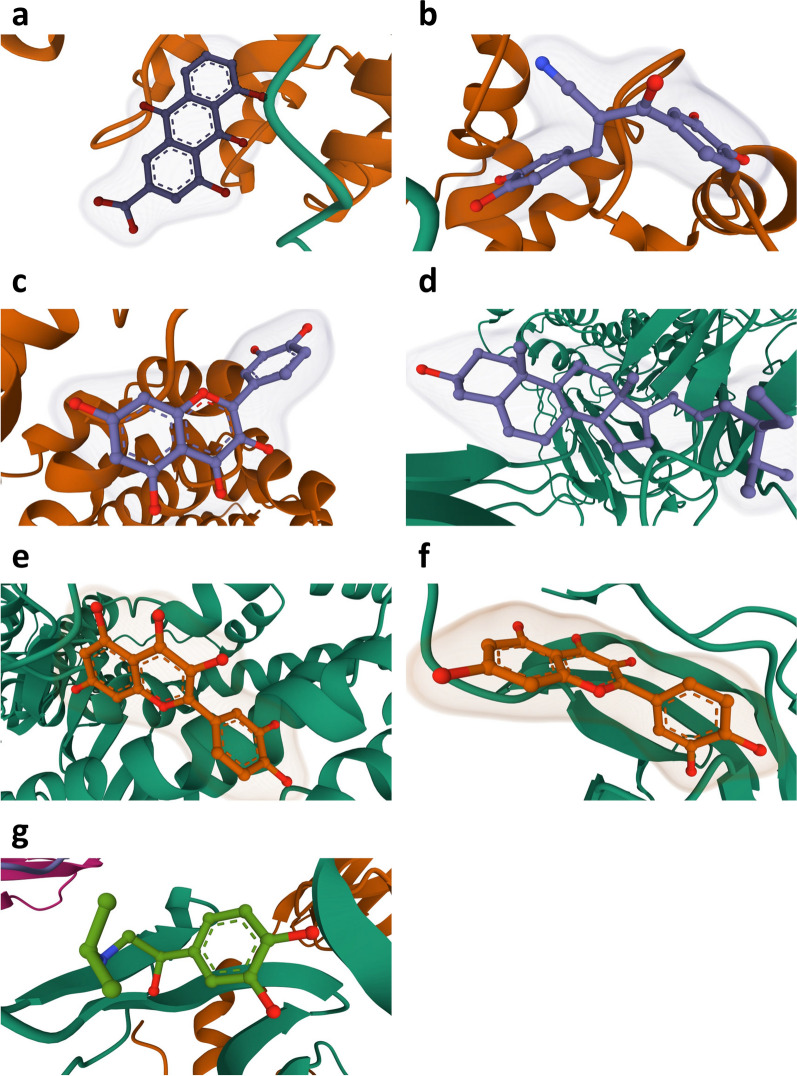
Docking results of available proteins small molecules. **a** FEN1 docking Rhein, **b** FEN1 docking Tyrphostin AG 538, **c** FEN1 docking Quercetin, **d** C5 docking Quercetin, **e** ATP2A1 docked to Quercetin, **f** IFNGR2 docked to Quercetin, **g** HLA-DPA1 docking (–)-isoprenaline

In order to assess the affinity of drug candidates for their targets and from this to understand the drug target's druggability, molecular docking was performed in this study. Autodock Vina v.1.2.2 was used to obtain the binding sites and interactions of the first five drug candidates with the proteins encoded by the corresponding genes and to generate the binding energy for each interaction, yielding valid docking results for a total of seven proteins with the drugs (Table 4 and Fig. 9). Each medication candidate connects to its protein target via visible hydrogen bonds and strong electrostatic interactions. In addition, the binding pocket of each target was successfully occupied by four drug candidates. C5 and Quercetin exhibited the lowest binding energy (− 9.364 kcal/mol), indicating extremely stable binding.

**Table 4:** Docking results of available proteins with small molecules

Target	PDB ID	Drug	PubChem ID	Binding energy
FEN1	3UVU	Rhein	10,168	− 6.699
FEN1	3UVU	Tyrphostin AG 538	5,328,760	− 7.252
FEN1	3UVU	Quercetin	5,280,343	− 7.046
C5	3CU7	Quercetin	5,280,343	− 9.364
ATP2A1	O14983 (EMBL-EBI)	Quercetin	5,280,343	− 7.789
IFNGR2	5EH1	Quercetin	5,280,343	− 5.71
HLA-DPA1	4P5K	(–)-isoprenaline	443,372	− 5.428

The lower the Binding Energy, the better the binding effect and the higher the affinity

## Discussion

This study identified seven drug targets for RA:, CCR6, HLA-DPA1,HLA-DRB1, IFNGR2, C5, ATP2A1and FEN1 based on several MR methods (Wald ratio/IVW, MR-Egger, weighted median method, horizontal multiplicity test and Cochran's Q heterogeneity test), which were able to exclude confounding factors of measurement and for measurement, while co-localization provided further strong evidence. To further illustrate the possible pleiotropy of the target genes and potential side effects of the drugs, phenome-wide association analysis was also employed. In addition, enrichment analysis and PPI networks were performed in this study in order to understand the biological significance of these drug targets. Finally, drugs corresponding to these targets were predicted and molecularly docked in this study, further demonstrating the druggable value of these target genes.

CCR6 is a G protein-coupled receptor found on a wide range of immune cells, including immature DCs [48, 49], innate lymphocytes, regulatory CD4 T cells (Tregs), Th17 cells [50, 51], and B cells [52]. CCR6 is implicated in cell migration in physiological and inflammatory circumstances, as well as in adaptive immunity [53, 54]. In a previous RA GWAS containing 41,282 individuals, the associated SNPs were in close proximity to known immune-functional genes including CCR6 [13]. Previous studies have also shown a significant reduction in arthritis severity and migration of Th17 cells to joints following the use of monoclonal antibodies against CCR6 [55]. HLA-DPA1 was identified as a common drug target for both Sjogren's syndrome and multiple sclerosis in a previous study [56], while another study more directly by weighted gene co-expression network (WGCNA) and linkage map (CMap) of illustrating HLA-DPA1 as a drug target for RA [57]. HLA-DRB1 is a recognised susceptibility-associated gene [58] with the strongest association with autoantibody-positive RA [59, 60] and the HLA-DRB1*13 allele was found to confer strong protection against RA [61], which is consistent with the results of this study.IFNGR2 is a known causally associated gene for RA [62] and has also been identified as a potential target for the autoimmune disease, psoriasis [63]. GWAS study found the chronic inflammation-associated gene C5 to be in linkage disequilibrium with RA-associated SNPs [64] and a second-generation monoclonal antibody drug against C5, Ravulizumab, has now been approved [65]. ATP2A1, a cancer-associated immunomarker, shows good affinity for HLC-018, a novel aniline-linked small molecule [66], indicating its potential as a drug target for the treatment of cancer [67]. These results suggest that the RA drug targets proposed in this study are strongly associated with RA and have a high medicinal value, promising the design of therapies against these genes for RA.

The present study has a number of significant advantages. First, this is the first study to use MR to identify RA drug targets, drawing on data from the largest publicly accessible RA risk GWAS to date. Furthermore, replication is not a regular technique in MR analysis [68], but this study replicated the MR results in two large cohorts, requiring genes to reach significance in both GWAS cohorts in order to be used as drug targets genes, validating the robustness of the results and greatly reducing the potential for false positives, which could further improve the success rate of clinical trials. The enrichment analysis illustrates the functional properties of these genes and the regulatory relationships of these drug target genes through PPI provided a potential possibility for the development of RA drugs through bypass. The final drug predictions illustrate the medicinal potential of these genes, and the high binding activity of molecular docking indicates the strong potential of these genes as drug targets. This study contains a complete evaluation from identification to drug binding properties, suggesting seven drug targets for RA with strong evidence.

### Limitations

The study is subject to several limitations that warrant consideration. Mendelian randomization (MR) offers valuable insights into causal relationships; however, it assumes low-dose drug exposure and a linear link between exposure and outcome, which may not fully replicate real-world clinical trials where high dosages are often evaluated over a short period. Consequently, the MR results may not precisely mirror the effect sizes observed in practical clinical settings and might not fully anticipate the impact of a drug [21].

Another limitation arises from the diversity of the study cohort. While eQTLs analysis includes individuals of non-European descent, the rheumatoid arthritis (RA) population consists solely of Europeans. This discrepancy in population backgrounds could introduce potential bias in the MR effect estimates, given the differences in genetic background and linkage disequilibrium patterns.

Moreover, the reliance on blood eQTLs for MR testing poses challenges in identifying the most effective tissue for treatment. Different tissues may have distinct genetic regulatory mechanisms, and solely focusing on blood eQTLs might not provide a comprehensive understanding of the disease and its potential treatments.

The study’s generalizability is constrained by its predominant inclusion of individuals of European descent. Extrapolating the findings to individuals of other ethnicities requires further research and validation to ensure the results’ broader applicability.

Despite rigorous efforts to minimize bias, MR analysis remains vulnerable to unmeasured factors or pleiotropy, which could influence the results. It is essential to acknowledge these limitations and their potential impact on the study’s conclusions.

Furthermore, the study primarily focused on cis-eQTLs and their relationship with rheumatoid arthritis, potentially overlooking other regulatory elements and environmental factors contributing to the complex nature of the disease.

Enrichment analysis, while valuable, has its inherent limitations as it relies on predefined gene sets or pathways, which might not encompass the full range of possible biological mechanisms or interactions. The absence of significant enrichment does not necessarily imply the absence of biological relevance, and researchers should interpret the results with caution.

Finally, the accuracy of molecular docking analysis heavily relies on the quality of the protein structures and ligands. While this approach identifies potential drug targets, it does not guarantee their effectiveness in clinical settings. Subsequent experimental validation and clinical trials are necessary to confirm the therapeutic potential of the identified targets.

Acknowledging and addressing these limitations will pave the way for future research to improve the understanding of rheumatoid arthritis and its potential treatments. Integrating diverse populations, omics data, and exploring alternative analytical approaches can contribute to a more comprehensive perspective and advance the field in meaningful ways.

### Recommendations for future explorations

In light of the study's limitations, several recommendations can guide future research in this crucial area. Firstly, future investigations should aim to bridge the gap between Mendelian randomization (MR) and real-world clinical trials by integrating high-dose short-term exposure experiments alongside MR analysis. This approach will improve the understanding of how drug effects observed in MR relate to clinical outcomes and enhance the translation of research findings into practical medical applications.

Secondly, to address potential bias and improve the generalizability of results, future studies should include diverse population cohorts representing various ethnic backgrounds. Validating findings in individuals with different genetic backgrounds can enhance the applicability of the research outcomes to a broader patient population.

Thirdly, integrating additional omics data and environmental factors in future studies can offer a more comprehensive understanding of the underlying mechanisms of rheumatoid arthritis. By considering various biological factors beyond genetic data, researchers can gain insights into the complex interplay of genetic, environmental, and lifestyle factors contributing to the disease.

Fourthly, to expand the understanding of rheumatoid arthritis pathogenesis, future investigations should explore regulatory elements beyond cis-eQTLs. Investigating other regulatory mechanisms and environmental factors can provide a more holistic view of the disease’s complexity and potentially reveal novel therapeutic targets.

Fifthly, researchers should be cautious in interpreting enrichment analysis results and consider alternative approaches to minimize bias. As enrichment analysis relies on predefined gene sets or pathways, future studies can explore broader biological networks and interactions to capture a more comprehensive range of biological mechanisms.

Sixthly, to improve the accuracy and reliability of molecular docking analysis, researchers should invest in enhancing the quality of protein structures and ligands used in the simulations. Availability of experimental data and higher-quality structural information will contribute to more reliable predictions of potential drug targets.

Lastly, it is crucial to emphasize that the identification of potential drug targets through MR and molecular docking does not guarantee their effectiveness in clinical settings. Therefore, further experimental validation and rigorous clinical trials are necessary to confirm the therapeutic potential of these targets and assess their safety and efficacy in real-world scenarios.

By incorporating these recommendations into future research endeavors, scientists can advance the understanding of rheumatoid arthritis, uncover new therapeutic possibilities, and ultimately improve patient outcomes in the field of autoimmune disorders.

## Conclusion

In conclusion, this research utilized MR analysis to identify potential drug targets for rheumatoid arthritis (RA). Seven drug targets were found to be significant in both cohorts and supported by colocalization analysis. These genes are associated with immune function and have the potential to be effective therapeutic targets for RA. Additionally, drug prediction and molecular docking were used to validate the medicinal value of these targets. The findings offer promising leads for more effective RA treatments, potentially reducing drug development costs and advancing personalized medicine approaches. This research makes a valuable contribution to the field, underscoring the significance of these identified targets in RA therapy. Further research and clinical trials on drugs targeting these genes are warranted.

### Supplementary Information

**Additional file 1**: **Table S1.** Results of the three Mendelian randomization methods in the discovery phase. **Table S2.** Results of MR Egger intercept horizontal pleiotropy test at the discovery stage level. **Table S3.** Results of heterogeneity test at the discovery phase. **Table S4.** Results of the three Mendelian randomization methods in the replication phase. **Table S5.** Results of MR Egger intercept horizontal pleiotropy test at the replication stage level. **Table S6.** Results of heterogeneity test at the replication phase. **Figure S1-S14.** PheWAS results for each gene.

## Data Availability

The datasets analysed during the current study are available in the in the following repositories: eQTLs data were obtained from eQTLGen Consortium (https://eqtlgen.org/); RA discovery data was from a previous study [14] and downloaded on GWAS Catalog (https://www.ebi.ac.uk/gwas/studies/GCST90132223); RA replication data was from FinnGen Release 8 (https://www.finngen.fi/en).

## Abbreviations

RA: Rheumatoid arthritis
GWAS: Genome-wide association studies
SNP: Single Nucleotide Polymorphism
MR: Mendelian randomization
eQTL: Expression quantitative trait loci
cis-eQTL: Cis-expression quantitative trait loci
PheWAS: Phenome-wide association study
IVW: Inverse variance weighted
GO: Gene Ontology
KEGG: Kyoto Encyclopedia of Genes and Genomes
PPI: Protein–protein interaction
