# Supplementary material for: Identification of potential drug targets for rheumatoid arthritis from genetic insights: a Mendelian randomization study
Source: J Transl Med. 2023 Sep 11;21:616. doi: 10.1186/s12967-023-04474-z (PMC10496392; doi:10.1186/s12967-023-04474-z)
Supplement: Supplementary file 1 — Additional file 1: Table S1. Results of the three Mendelian randomization methods in the discovery phase. Table S2. Results of MR Egger intercept horizontal pleiotropy test at the discovery stage level. Table S3. Results of heterogeneity test at the discovery phase. Table S4. Results of the three Mendelian randomization methods in the replication phase. Table S5. Results of MR Egger intercept horizontal pleiotropy test at the replication stage level. Table S6. Results of heterogeneity test at the replication phase. Figure S1-S14. PheWAS results for each gene. [file 12967_2023_4474_MOESM1_ESM.docx]

**Additional file list**

Table S1. Results of the three Mendelian randomization methods in the discovery phase.

Table S2. Results of MR Egger intercept horizontal pleiotropy test at the discovery stage level

Table S3. Results of heterogeneity test at the discovery phase.

Table S4. Results of the three Mendelian randomization methods in the replication phase.

Table S5. Results of MR Egger intercept horizontal pleiotropy test at the replication stage level

Table S6. Results of heterogeneity test at the replication phase.

**Figure S1-S14. PheWAS results for each gene.**

Table S1. Results of the three Mendelian randomization methods in the discovery phase.

| Gene | Methods | No.SNPs | Beta | SE | P Value |
| --- | --- | --- | --- | --- | --- |
| ANTXR2 | MR Egger | 16 | -0.12636 | 0.07913 | 0.132609 |
| ANTXR2 | Weighted median | 16 | -0.19622 | 0.051499 | 0.000139 |
| ANTXR2 | IVW | 16 | -0.17462 | 0.038879 | 7.08E-06 |
| APP | MR Egger | 21 | 0.078384 | 0.103002 | 0.456002 |
| APP | Weighted median | 21 | 0.181168 | 0.059953 | 0.002512 |
| APP | IVW | 21 | 0.238295 | 0.045501 | 1.63E-07 |
| ATP2A1 | IVW | 2 | 0.607882 | 0.13608 | 7.93E-06 |
| BRSK1 | MR Egger | 5 | 0.065398 | 0.460402 | 0.896047 |
| BRSK1 | Weighted median | 5 | -0.25737 | 0.096046 | 0.007371 |
| BRSK1 | IVW | 5 | -0.33664 | 0.074662 | 6.52E-06 |
| C5 | MR Egger | 21 | -0.06275 | 0.086034 | 0.474656 |
| C5 | Weighted median | 21 | -0.09412 | 0.027754 | 0.000696 |
| C5 | IVW | 21 | -0.10751 | 0.02508 | 1.81E-05 |
| CCR6 | MR Egger | 16 | 0.539383 | 0.094837 | 5.61E-05 |
| CCR6 | Weighted median | 16 | 0.50607 | 0.065593 | 1.21E-14 |
| CCR6 | IVW | 16 | 0.4029 | 0.054052 | 9.06E-14 |
| CD226 | MR Egger | 31 | -0.02262 | 0.058786 | 0.703267 |
| CD226 | Weighted median | 31 | -0.15613 | 0.036704 | 2.10E-05 |
| CD226 | IVW | 31 | -0.17504 | 0.028449 | 7.62E-10 |
| CD28 | IVW | 2 | 0.706546 | 0.158581 | 8.37E-06 |
| CDK4 | Wald ratio | 1 | -1.30714 | 0.298156 | 1.16E-05 |
| CTLA4 | MR Egger | 5 | -0.58647 | 0.976461 | 0.590448 |
| CTLA4 | Weighted median | 5 | -0.4832 | 0.112742 | 1.82E-05 |
| CTLA4 | IVW | 5 | -0.62627 | 0.111769 | 2.10E-08 |
| CYP21A2 | Wald ratio | 1 | 0.45379 | 0.06338 | 8.08E-13 |
| DNMT3A | MR Egger | 9 | -0.43661 | 0.261043 | 0.138333 |
| DNMT3A | Weighted median | 9 | -0.37642 | 0.128494 | 0.003395 |
| DNMT3A | IVW | 9 | -0.45764 | 0.096719 | 2.23E-06 |
| FCGR2B | MR Egger | 21 | 0.133806 | 0.039844 | 0.003302 |
| FCGR2B | Weighted median | 21 | 0.09758 | 0.025495 | 0.000129 |
| FCGR2B | IVW | 21 | 0.103828 | 0.02037 | 3.45E-07 |
| FEN1 | Wald ratio | 1 | 1.944697 | 0.372785 | 1.82E-07 |
| GAK | MR Egger | 6 | -0.0227 | 0.291178 | 0.941594 |
| GAK | Weighted median | 6 | -0.44411 | 0.149249 | 0.002924 |
| GAK | IVW | 6 | -0.47916 | 0.110262 | 1.39E-05 |
| GPNMB | MR Egger | 10 | -0.11704 | 0.094492 | 0.250606 |
| GPNMB | Weighted median | 10 | -0.11885 | 0.032075 | 0.000211 |
| GPNMB | IVW | 10 | -0.12711 | 0.027856 | 5.03E-06 |
| HLA-DPA1 | MR Egger | 4 | 0.260747 | 0.090807 | 0.1029 |
| HLA-DPA1 | Weighted median | 4 | 0.392726 | 0.020296 | 2.06E-83 |
| HLA-DPA1 | IVW | 4 | 0.412114 | 0.042424 | 2.62E-22 |
| HLA-DRB1 | Wald ratio | 1 | -0.91368 | 0.029095 | 1.85E-216 |
| HLCS | MR Egger | 39 | -0.06307 | 0.052786 | 0.239775 |
| HLCS | Weighted median | 39 | -0.09484 | 0.034767 | 0.006373 |
| HLCS | IVW | 39 | -0.1077 | 0.023363 | 4.02E-06 |
| HSPA1B | Wald ratio | 1 | 1.177701 | 0.104664 | 2.26E-29 |
| IFNGR2 | MR Egger | 17 | -0.19952 | 0.043097 | 0.000327 |
| IFNGR2 | Weighted median | 17 | -0.16884 | 0.024551 | 6.11E-12 |
| IFNGR2 | IVW | 17 | -0.17077 | 0.020903 | 3.09E-16 |
| ITGA9 | MR Egger | 23 | 0.28827 | 0.11857 | 0.02409 |
| ITGA9 | Weighted median | 23 | 0.208665 | 0.057686 | 0.000298 |
| ITGA9 | IVW | 23 | 0.234941 | 0.041333 | 1.31E-08 |
| ITPR3 | MR Egger | 27 | -0.32213 | 0.143901 | 0.034327 |
| ITPR3 | Weighted median | 27 | -0.23358 | 0.030993 | 4.82E-14 |
| ITPR3 | IVW | 27 | -0.25617 | 0.057732 | 9.11E-06 |
| LGALS9 | MR Egger | 16 | 0.073046 | 0.033853 | 0.048806 |
| LGALS9 | Weighted median | 16 | 0.066431 | 0.019089 | 0.000501 |
| LGALS9 | IVW | 16 | 0.071545 | 0.015812 | 6.05E-06 |
| MAST2 | MR Egger | 7 | 0.175848 | 0.226825 | 0.473241 |
| MAST2 | Weighted median | 7 | 0.392007 | 0.105839 | 0.000212 |
| MAST2 | IVW | 7 | 0.405627 | 0.093559 | 1.45E-05 |
| MMEL1 | MR Egger | 13 | 0.005057 | 0.072804 | 0.945871 |
| MMEL1 | Weighted median | 13 | 0.179664 | 0.025958 | 4.48E-12 |
| MMEL1 | IVW | 13 | 0.163914 | 0.034959 | 2.75E-06 |
| NOTCH4 | Wald ratio | 1 | 0.893053 | 0.157564 | 1.45E-08 |
| OPRL1 | MR Egger | 9 | -0.14981 | 0.138931 | 0.316648 |
| OPRL1 | Weighted median | 9 | -0.14201 | 0.035568 | 6.54E-05 |
| OPRL1 | IVW | 9 | -0.1324 | 0.030936 | 1.87E-05 |
| PAM | MR Egger | 35 | -0.05642 | 0.033972 | 0.106229 |
| PAM | Weighted median | 35 | -0.07081 | 0.014348 | 8.02E-07 |
| PAM | IVW | 35 | -0.06811 | 0.01138 | 2.17E-09 |
| PSMB7 | IVW | 2 | -0.50319 | 0.103329 | 1.12E-06 |
| SIGLEC6 | MR Egger | 5 | -0.24957 | 0.260662 | 0.408968 |
| SIGLEC6 | Weighted median | 5 | -0.35222 | 0.08529 | 3.63E-05 |
| SIGLEC6 | IVW | 5 | -0.383 | 0.070977 | 6.81E-08 |
| TSSK6 | Wald ratio | 1 | 0.50027 | 0.10524 | 2.00E-06 |

Table S2. Results of MR Egger intercept horizontal pleiotropy test at the discovery stage level

| Gene | Intercept | SE | P Value |
| --- | --- | --- | --- |
| CTLA4 | -0.00547 | 0.133114 | 0.969781 |
| MMEL1 | 0.068101 | 0.028509 | 0.035941 |
| MAST2 | 0.02271 | 0.020422 | 0.316715 |
| APP | 0.022155 | 0.012802 | 0.099746 |
| HLA-DPA1 | 0.116093 | 0.065096 | 0.216455 |
| PAM | -0.0072 | 0.019678 | 0.716929 |
| HLCS | -0.01079 | 0.011446 | 0.351785 |
| CD226 | -0.02999 | 0.010359 | 0.007128 |
| OPRL1 | 0.00601 | 0.046642 | 0.901096 |
| ANTXR2 | -0.0101 | 0.014419 | 0.495312 |
| LGALS9 | -0.00078 | 0.015493 | 0.960513 |
| GPNMB | -0.00356 | 0.031928 | 0.913884 |
| DNMT3A | -0.00156 | 0.018039 | 0.933321 |
| FCGR2B | -0.01379 | 0.015724 | 0.391306 |
| CCR6 | -0.02108 | 0.012361 | 0.110137 |
| ITGA9 | -0.00657 | 0.013693 | 0.636284 |
| GAK | -0.04526 | 0.026719 | 0.16556 |
| ITPR3 | 0.024254 | 0.048328 | 0.620163 |
| IFNGR2 | 0.012821 | 0.016803 | 0.457295 |
| SIGLEC6 | -0.01606 | 0.029999 | 0.629448 |
| C5 | -0.01618 | 0.029699 | 0.592218 |

Table S3. Results of heterogeneity test at the discovery phase

| Gene | Methods | Q Value | Q df | P Value |
| --- | --- | --- | --- | --- |
| ITPR3 | IVW | 532.9502 | 26 | 5.23E-96 |
| ITPR3 | MR Egger | 527.6347 | 25 | 1.42E-95 |
| HLA-DPA1 | IVW | 17.30186 | 3 | 0.000613 |
| C5 | MR Egger | 42.42904 | 19 | 0.001551 |
| C5 | IVW | 43.09188 | 20 | 0.001988 |
| MMEL1 | IVW | 29.23627 | 12 | 0.003634 |
| CTLA4 | MR Egger | 12.83029 | 3 | 0.005018 |
| CTLA4 | IVW | 12.83752 | 4 | 0.012098 |
| HLA-DPA1 | MR Egger | 6.679496 | 2 | 0.035446 |
| MMEL1 | MR Egger | 19.25038 | 11 | 0.056745 |
| CCR6 | IVW | 23.04562 | 15 | 0.083176 |
| PAM | MR Egger | 42.134 | 33 | 0.132402 |
| FCGR2B | MR Egger | 25.69854 | 19 | 0.13881 |
| FCGR2B | IVW | 26.73944 | 20 | 0.142755 |
| PAM | IVW | 42.30475 | 34 | 0.155162 |
| CCR6 | MR Egger | 19.08036 | 14 | 0.161901 |
| CD226 | IVW | 35.47713 | 30 | 0.225694 |
| CD28 | IVW | 1.22813 | 1 | 0.267771 |
| SIGLEC6 | MR Egger | 3.494629 | 3 | 0.321459 |
| PSMB7 | IVW | 0.927918 | 1 | 0.335404 |
| OPRL1 | MR Egger | 7.705909 | 7 | 0.359239 |
| LGALS9 | MR Egger | 14.50871 | 14 | 0.412539 |
| SIGLEC6 | IVW | 3.828689 | 4 | 0.429686 |
| OPRL1 | IVW | 7.724187 | 8 | 0.460866 |
| APP | IVW | 19.93938 | 20 | 0.461728 |
| LGALS9 | IVW | 14.51135 | 15 | 0.487153 |
| HLCS | MR Egger | 36.16993 | 37 | 0.507772 |
| HLCS | IVW | 37.05923 | 38 | 0.512801 |
| ANTXR2 | MR Egger | 13.15423 | 14 | 0.514417 |
| ATP2A1 | IVW | 0.406904 | 1 | 0.523545 |
| GAK | IVW | 3.995711 | 5 | 0.550034 |
| ANTXR2 | IVW | 13.64442 | 15 | 0.55265 |
| BRSK1 | MR Egger | 2.091199 | 3 | 0.553695 |
| CD226 | MR Egger | 27.0944 | 29 | 0.566612 |
| BRSK1 | IVW | 2.874341 | 4 | 0.57907 |
| APP | MR Egger | 16.94467 | 19 | 0.593617 |
| MAST2 | IVW | 3.574965 | 6 | 0.733971 |
| DNMT3A | MR Egger | 4.07695 | 7 | 0.770873 |
| MAST2 | MR Egger | 2.338358 | 5 | 0.800617 |
| IFNGR2 | MR Egger | 9.653961 | 15 | 0.840924 |
| DNMT3A | IVW | 4.084471 | 8 | 0.849423 |
| IFNGR2 | IVW | 10.23613 | 16 | 0.854009 |
| GPNMB | MR Egger | 3.830347 | 8 | 0.872096 |
| GAK | MR Egger | 1.126856 | 4 | 0.889988 |
| GPNMB | IVW | 3.842803 | 9 | 0.921451 |
| ITGA9 | MR Egger | 11.94748 | 21 | 0.941025 |
| ITGA9 | IVW | 12.17775 | 22 | 0.953599 |

Table S4. Results of the three Mendelian randomization methods in the replication phase.

| Gene | Methods | No.SNPs | Beta | SE | P Value |
| --- | --- | --- | --- | --- | --- |
| ATP2A1 | IVW | 2 | 0.823317 | 0.169218 | 1.14E-06 |
| C5 | MR Egger | 20 | -0.06638 | 0.0674 | 0.337725 |
| C5 | Weighted median | 20 | -0.14064 | 0.026469 | 1.08E-07 |
| C5 | IVW | 20 | -0.12278 | 0.019135 | 1.39E-10 |
| CCR6 | MR Egger | 16 | 0.609868 | 0.131378 | 0.000381 |
| CCR6 | Weighted median | 16 | 0.541994 | 0.073855 | 2.16E-13 |
| CCR6 | IVW | 16 | 0.373677 | 0.075262 | 6.87E-07 |
| CTLA4 | MR Egger | 4 | 0.018632 | 1.5952 | 0.991741 |
| CTLA4 | Weighted median | 4 | -0.65166 | 0.157539 | 3.53E-05 |
| CTLA4 | IVW | 4 | -0.69408 | 0.188987 | 0.00024 |
| FCGR2B | MR Egger | 20 | 0.112895 | 0.049665 | 0.035504 |
| FCGR2B | Weighted median | 20 | 0.122985 | 0.026914 | 4.89E-06 |
| FCGR2B | IVW | 20 | 0.133333 | 0.025303 | 1.37E-07 |
| FEN1 | Wald ratio | 1 | 2.124276 | 0.545901 | 9.97E-05 |
| HLA-DPA1 | MR Egger | 4 | 0.127964 | 0.074185 | 0.226681 |
| HLA-DPA1 | Weighted median | 4 | 0.306453 | 0.028923 | 3.12E-26 |
| HLA-DPA1 | IVW | 4 | 0.326198 | 0.046162 | 1.59E-12 |
| HLA-DRB1 | MR Egger | 5 | -0.31091 | 0.694482 | 0.684731 |
| HLA-DRB1 | Weighted median | 5 | -0.41914 | 0.047675 | 1.48E-18 |
| HLA-DRB1 | IVW | 5 | -0.5122 | 0.145203 | 0.00042 |
| HLCS | MR Egger | 42 | 0.118136 | 0.046523 | 0.015099 |
| HLCS | Weighted median | 42 | 0.085206 | 0.031705 | 0.0072 |
| HLCS | IVW | 42 | 0.067785 | 0.02096 | 0.001221 |
| IFNGR2 | MR Egger | 19 | -0.12639 | 0.041933 | 0.007817 |
| IFNGR2 | Weighted median | 19 | -0.07331 | 0.026207 | 0.005155 |
| IFNGR2 | IVW | 19 | -0.06857 | 0.020249 | 0.000708 |
| ITPR3 | MR Egger | 27 | -0.20533 | 0.206121 | 0.328719 |
| ITPR3 | Weighted median | 27 | -0.29446 | 0.043842 | 1.86E-11 |
| ITPR3 | IVW | 27 | -0.28573 | 0.08269 | 0.000549 |
| OPRL1 | MR Egger | 8 | -0.34406 | 0.115889 | 0.024994 |
| OPRL1 | Weighted median | 8 | -0.13297 | 0.035448 | 0.000176 |
| OPRL1 | IVW | 8 | -0.12019 | 0.031268 | 0.000121 |

Table S5. Results of MR Egger intercept horizontal pleiotropy test at the replication stage level

| Gene | Intercept | SE | P Value |
| --- | --- | --- | --- |
| HLA-DRB1 | -0.15643 | 0.524219 | 0.784864 |
| HLA-DPA1 | 0.153152 | 0.05364 | 0.1039 |
| C5 | -0.02153 | 0.024666 | 0.39433 |
| OPRL1 | 0.075527 | 0.037993 | 0.093988 |
| HLCS | -0.01236 | 0.010194 | 0.232511 |
| CCR6 | -0.0344 | 0.016377 | 0.054295 |
| IFNGR2 | 0.024929 | 0.015833 | 0.133796 |
| FCGR2B | 0.009425 | 0.019562 | 0.635751 |
| ITPR3 | -0.02977 | 0.069695 | 0.672912 |

Table S6. Results of heterogeneity test at the replication phase.

| Gene | Methods | Q Value | Q df | P Value |
| --- | --- | --- | --- | --- |
| HLA-DRB1 | MR Egger | 684.6334 | 3 | 4.51E-148 |
| HLA-DRB1 | IVW | 704.9555 | 4 | 2.95E-151 |
| HLA-DPA1 | MR Egger | 1.209811 | 2 | 0.546126 |
| HLA-DPA1 | IVW | 9.36182 | 3 | 0.024848 |
| C5 | MR Egger | 10.42776 | 18 | 0.917046 |
| C5 | IVW | 11.18933 | 19 | 0.917305 |
| ATP2A1 | IVW | 0.815084 | 1 | 0.366621 |
| OPRL1 | MR Egger | 5.198683 | 6 | 0.518595 |
| OPRL1 | IVW | 9.150466 | 7 | 0.242033 |
| CTLA4 | MR Egger | 12.28836 | 2 | 0.002146 |
| CTLA4 | IVW | 13.53874 | 3 | 0.003605 |
| HLCS | MR Egger | 33.08064 | 40 | 0.772645 |
| HLCS | IVW | 34.55031 | 41 | 0.751347 |
| CCR6 | MR Egger | 25.34462 | 14 | 0.031314 |
| CCR6 | IVW | 33.33109 | 15 | 0.004223 |
| IFNGR2 | MR Egger | 11.59525 | 17 | 0.824003 |
| IFNGR2 | IVW | 14.07432 | 18 | 0.724234 |
| FCGR2B | MR Egger | 38.50571 | 18 | 0.003318 |
| FCGR2B | IVW | 39.00229 | 19 | 0.004414 |
| ITPR3 | MR Egger | 665.1171 | 25 | 2.83E-124 |
| ITPR3 | IVW | 669.9718 | 26 | 1.42E-124 |

Figure S1-S14. Results of PheWAS analysis for 7 genes. The bottom dashed line represents the Suggestive line and the top dashed line is the Significant line. Traits that exceeded the significant line were considered to be significantly associated with a gene. As can be seen, there were no significantly associated traits for any of the genes except ATP2A1, and information on traits significantly associated with ATP2A1 is shown in Table S7.


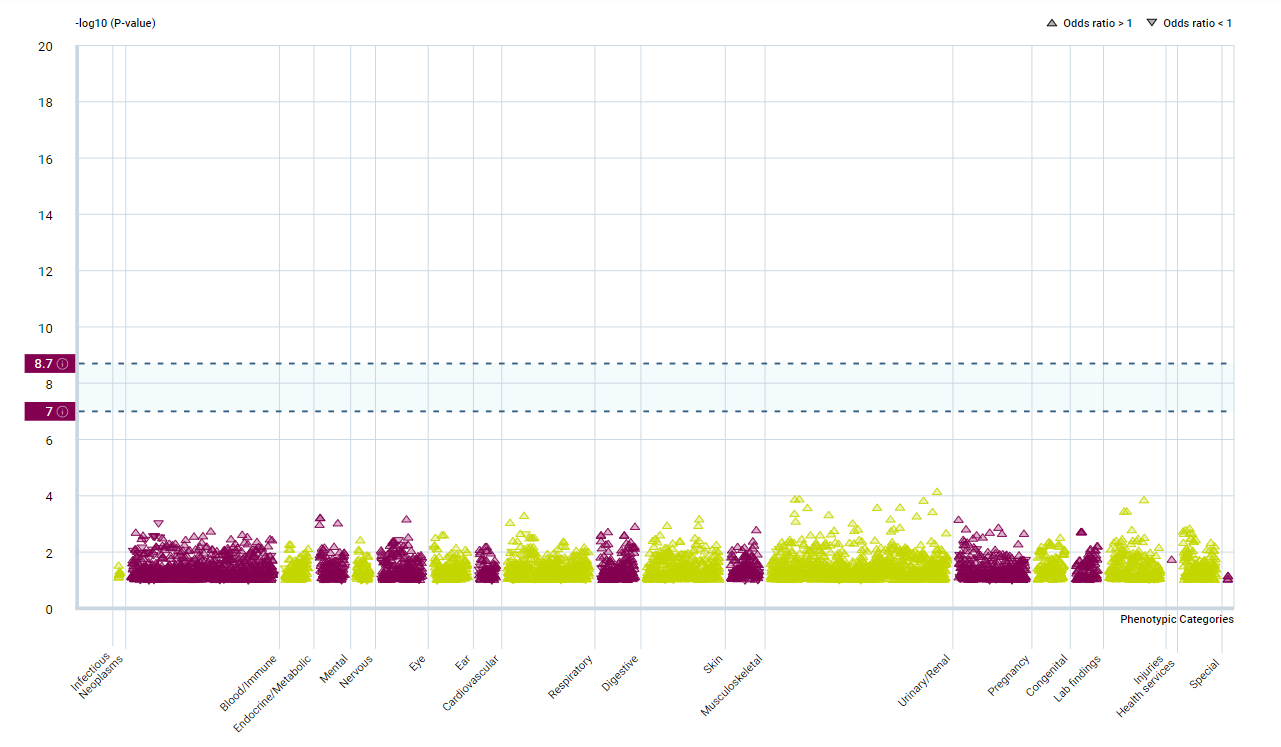


Figure S1.Binary traits PheWAS association with CCR6


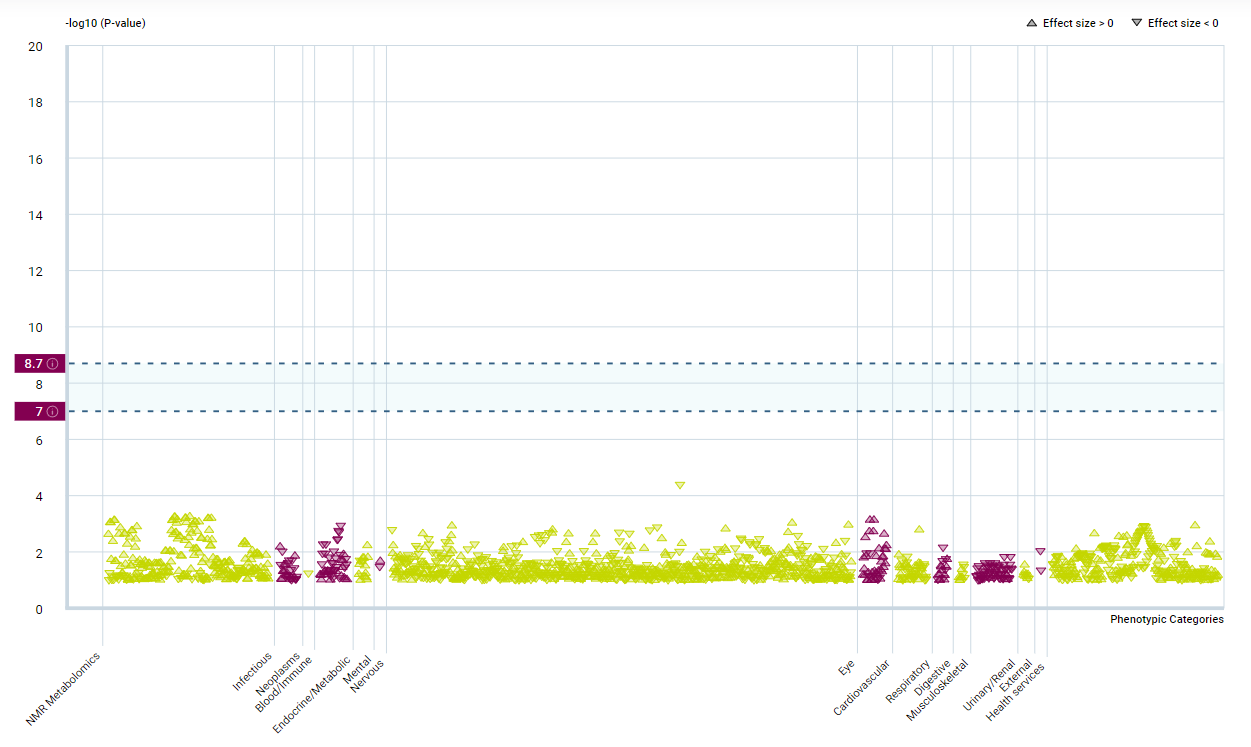


Figure S2. Continuous traits PheWAS association with CCR6


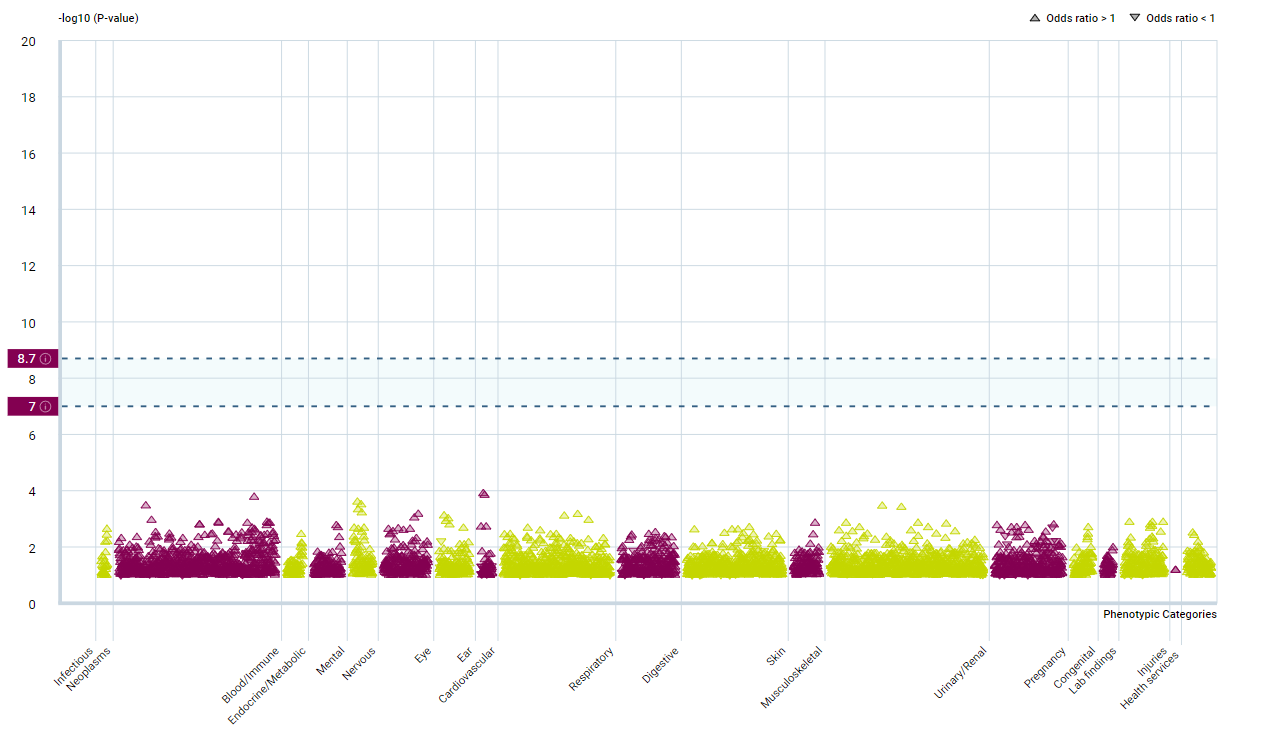
 Figure S3. Binary traits PheWAS association with HLA-DPA1


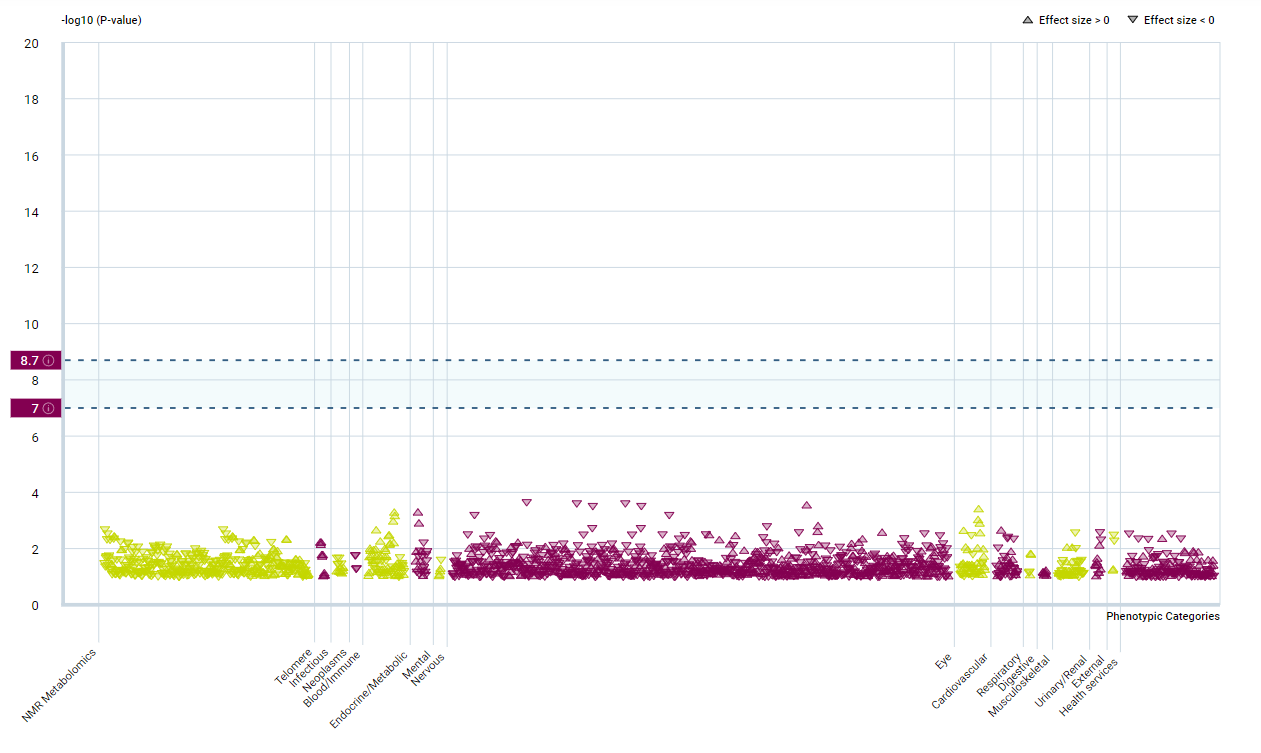


Figure S4. Continuous traits PheWAS association with HLA-DPA1


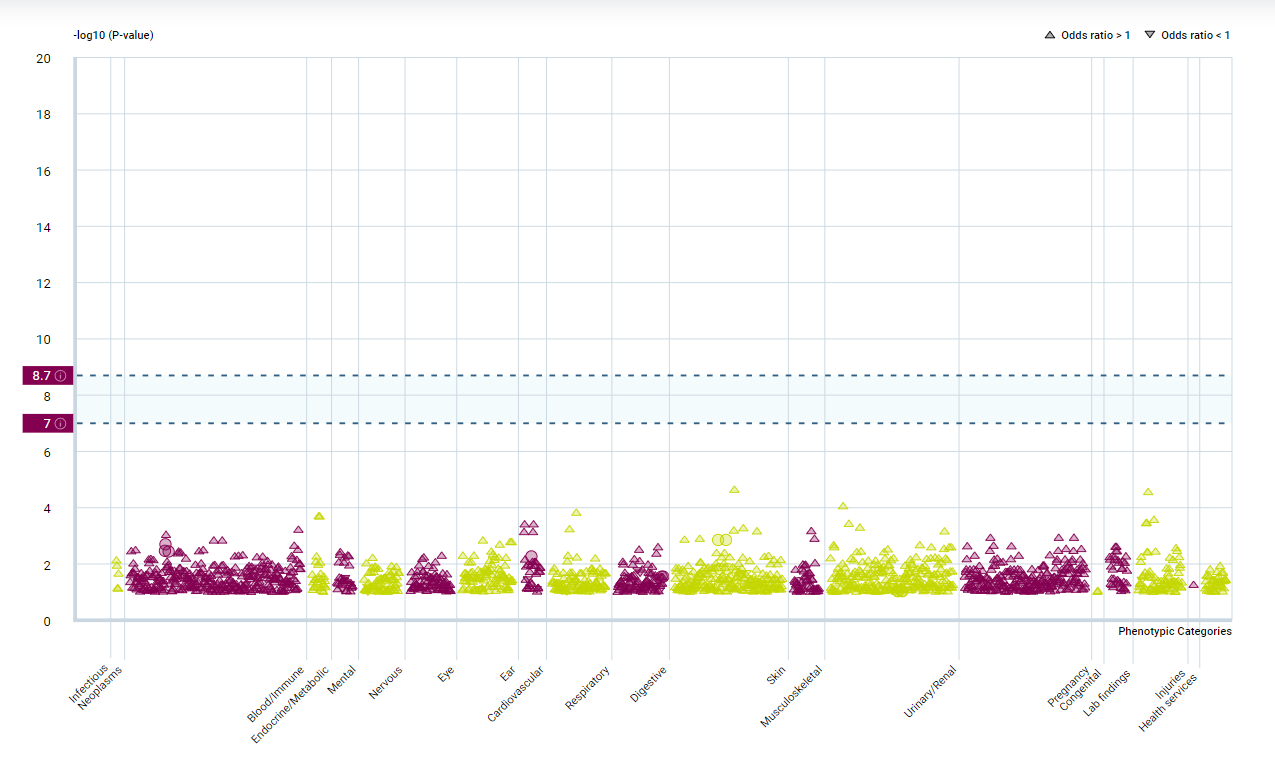


Figure S5. Binary traits PheWAS association with HLA-DRB1


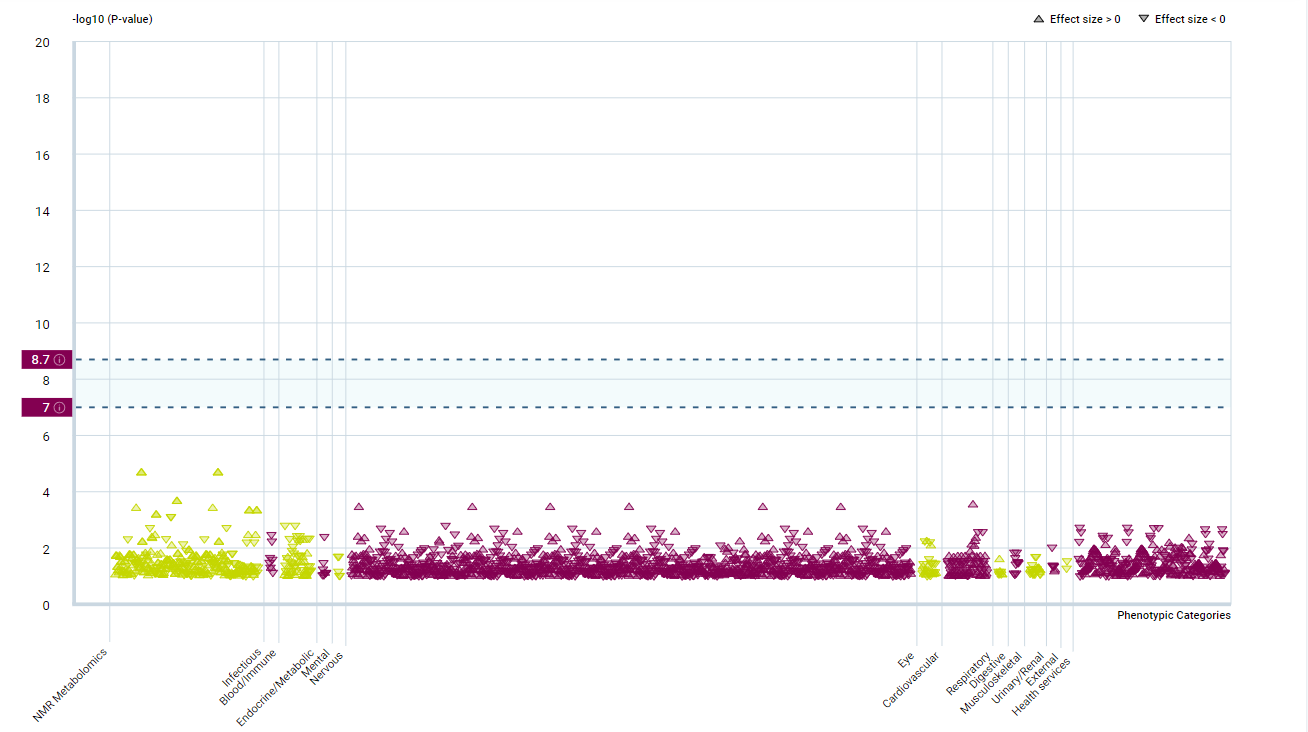


Figure S6. Continuous traits PheWAS association with HLA-DRB1


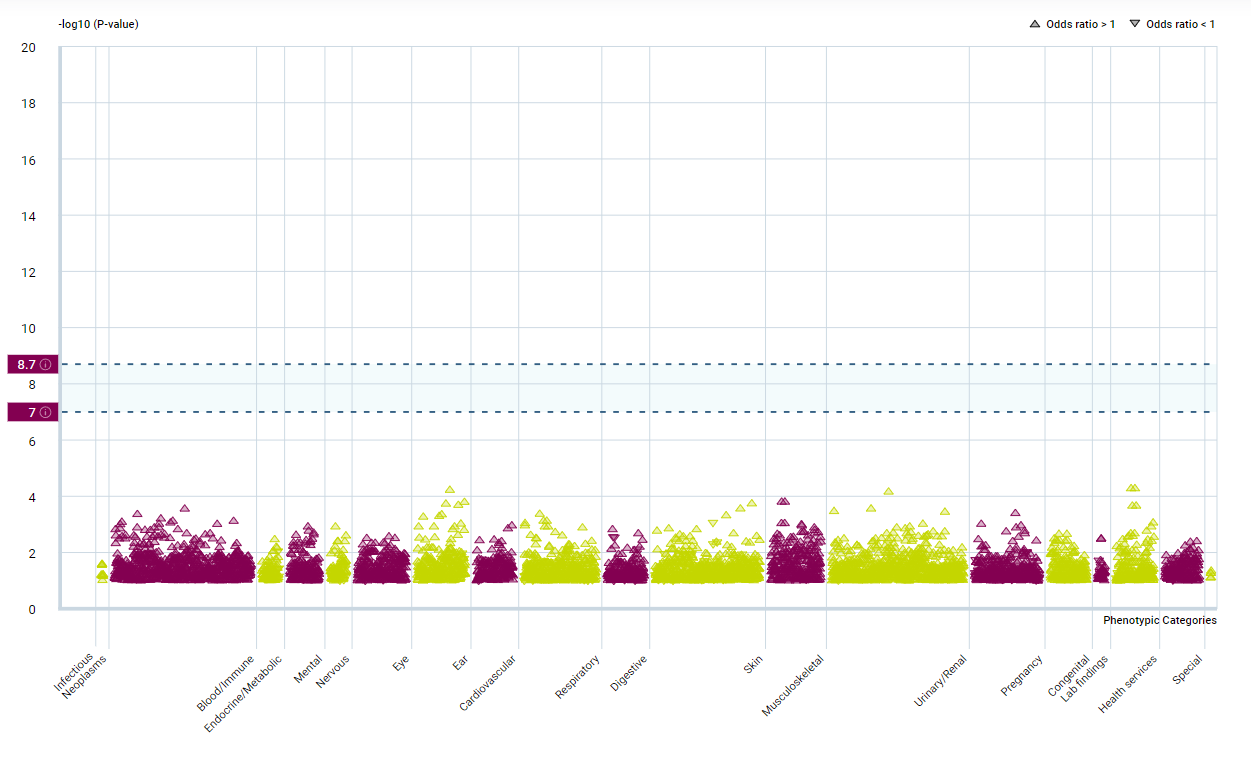


Figure S7. Binary traits PheWAS association with IFNGR2


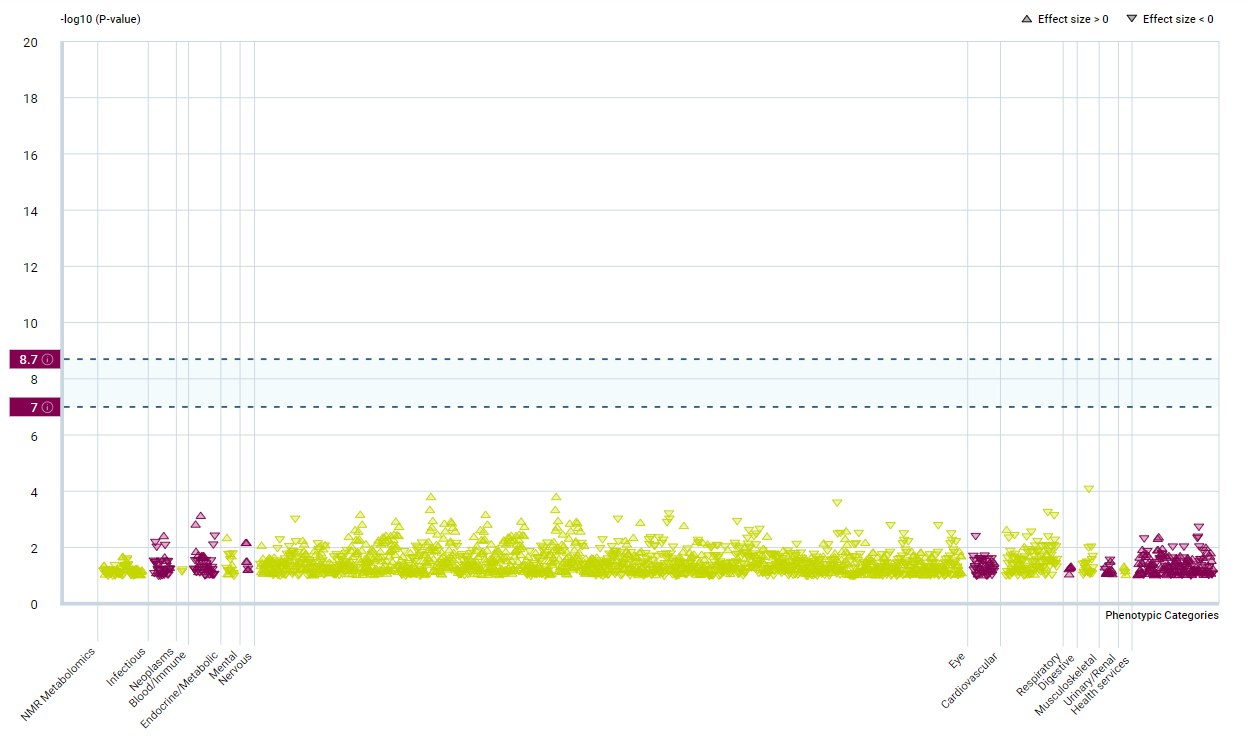


Figure S8. Continuous traits PheWAS association with IFNGR2


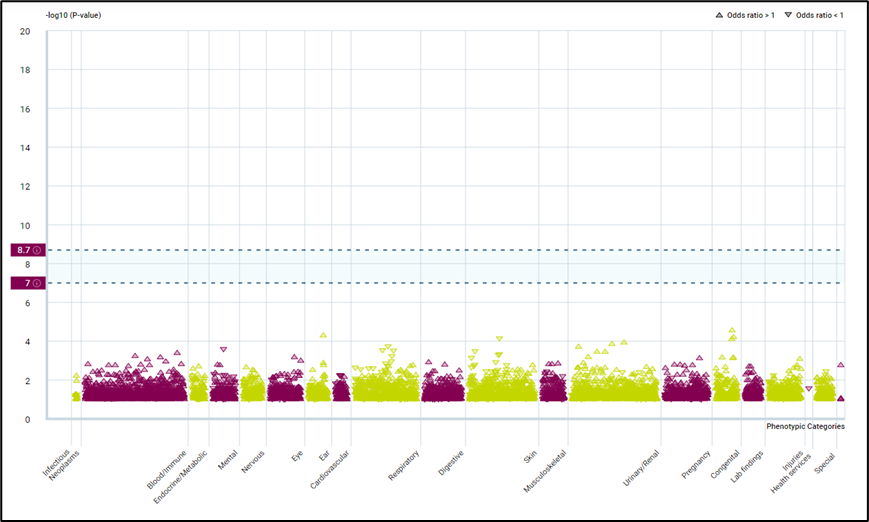
Figure S9. Binary traits PheWAS association with C5
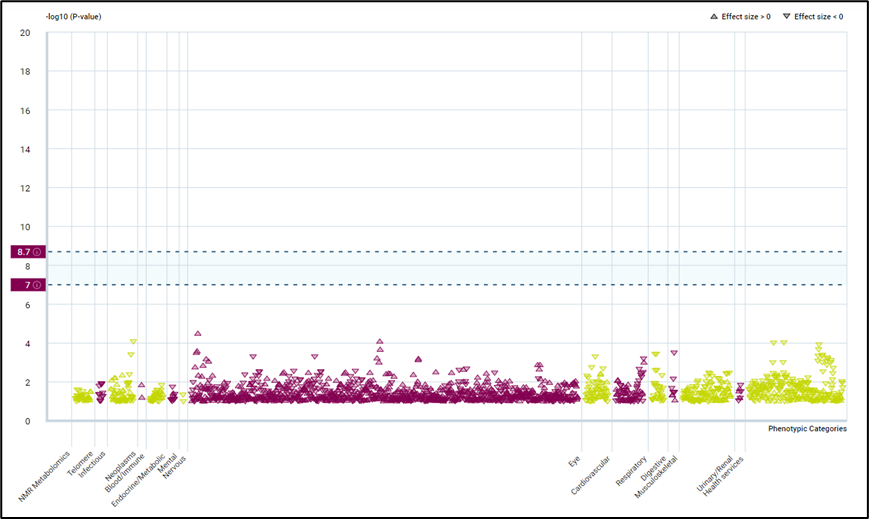


Figure S10. Continuous traits PheWAS association with C5


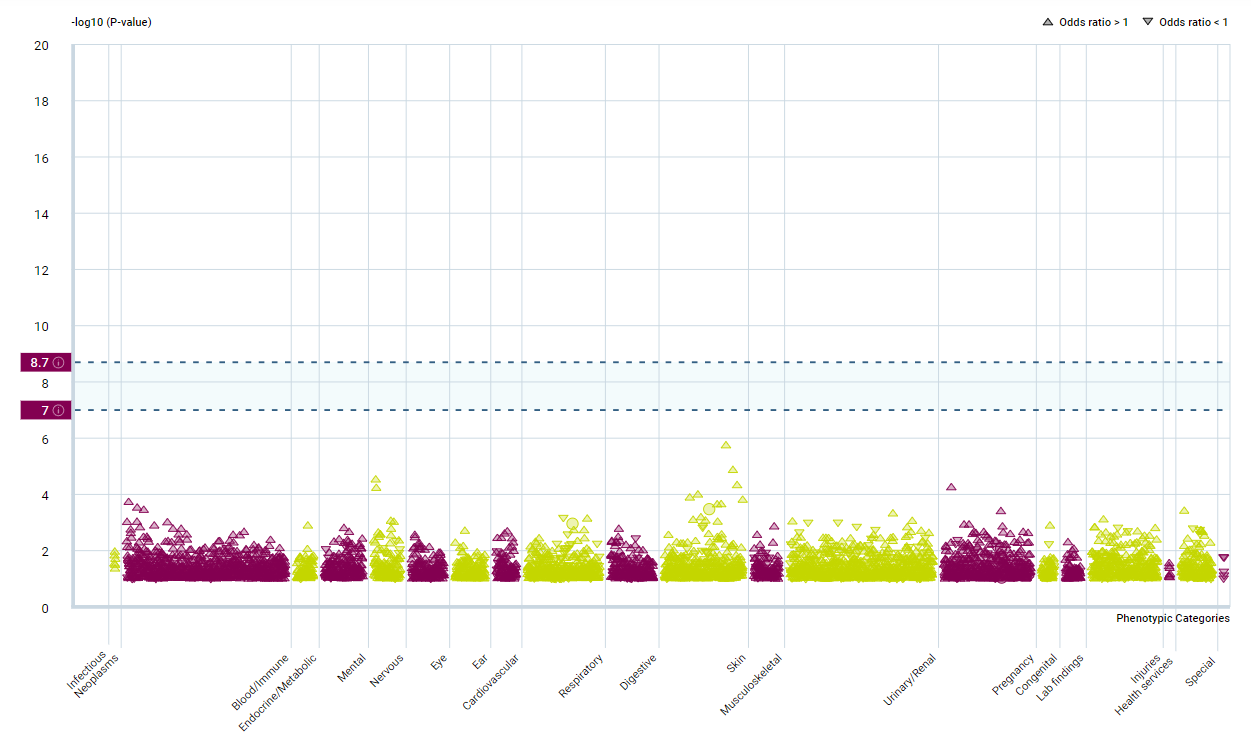


Figure S11. Binary traits PheWAS association with FEN1


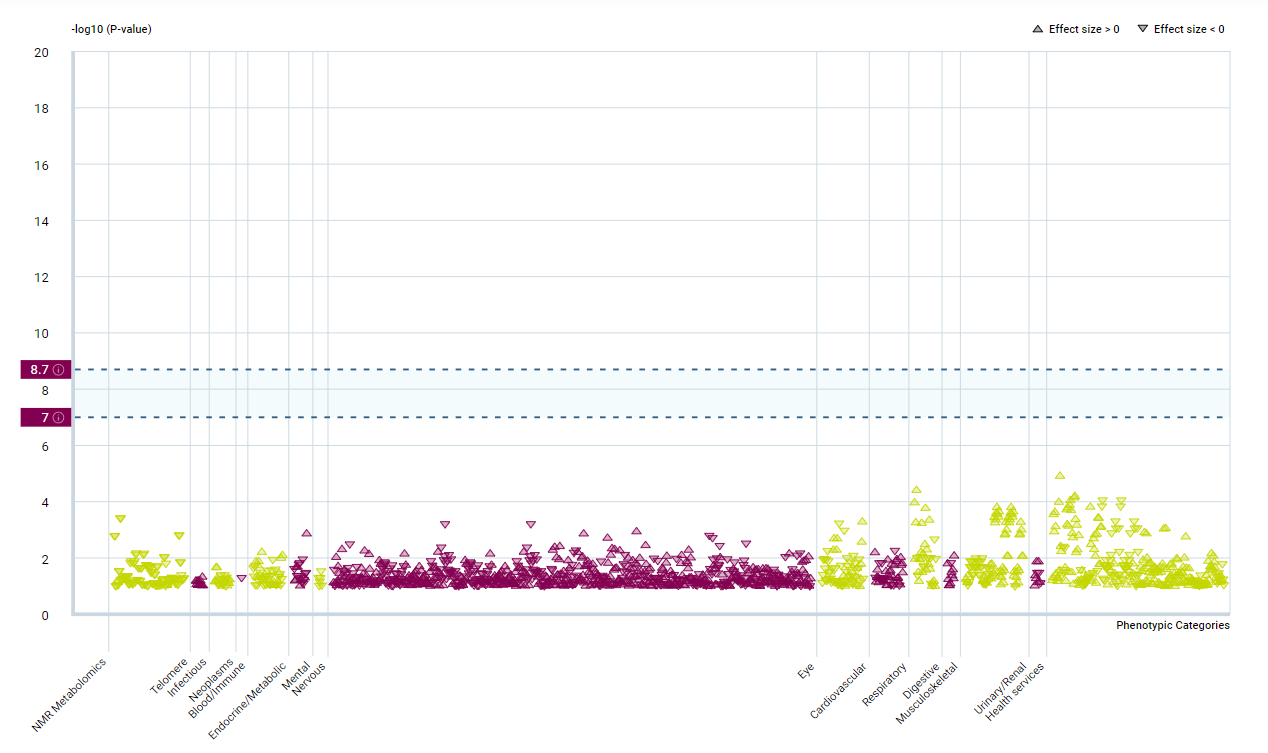
 Figure S12. Continuous traits PheWAS association with FEN1


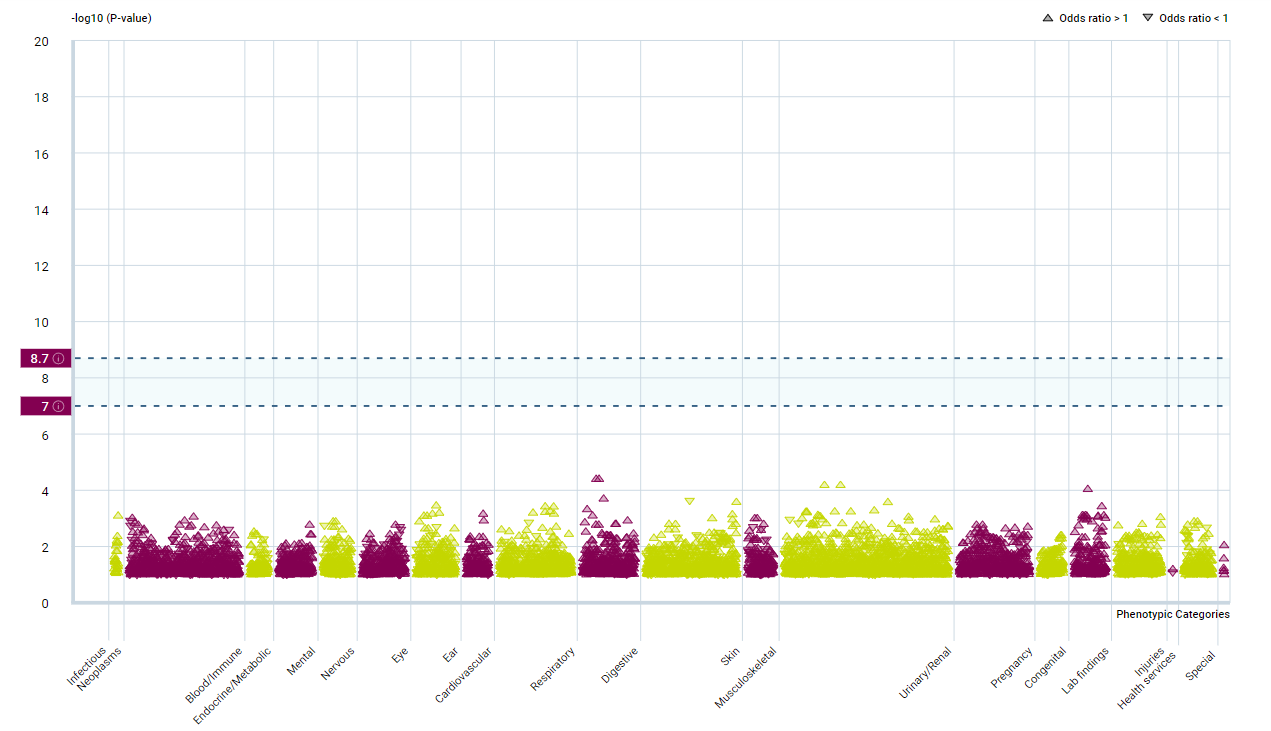


Figure S13. Binary traits PheWAS association with ATP2A1


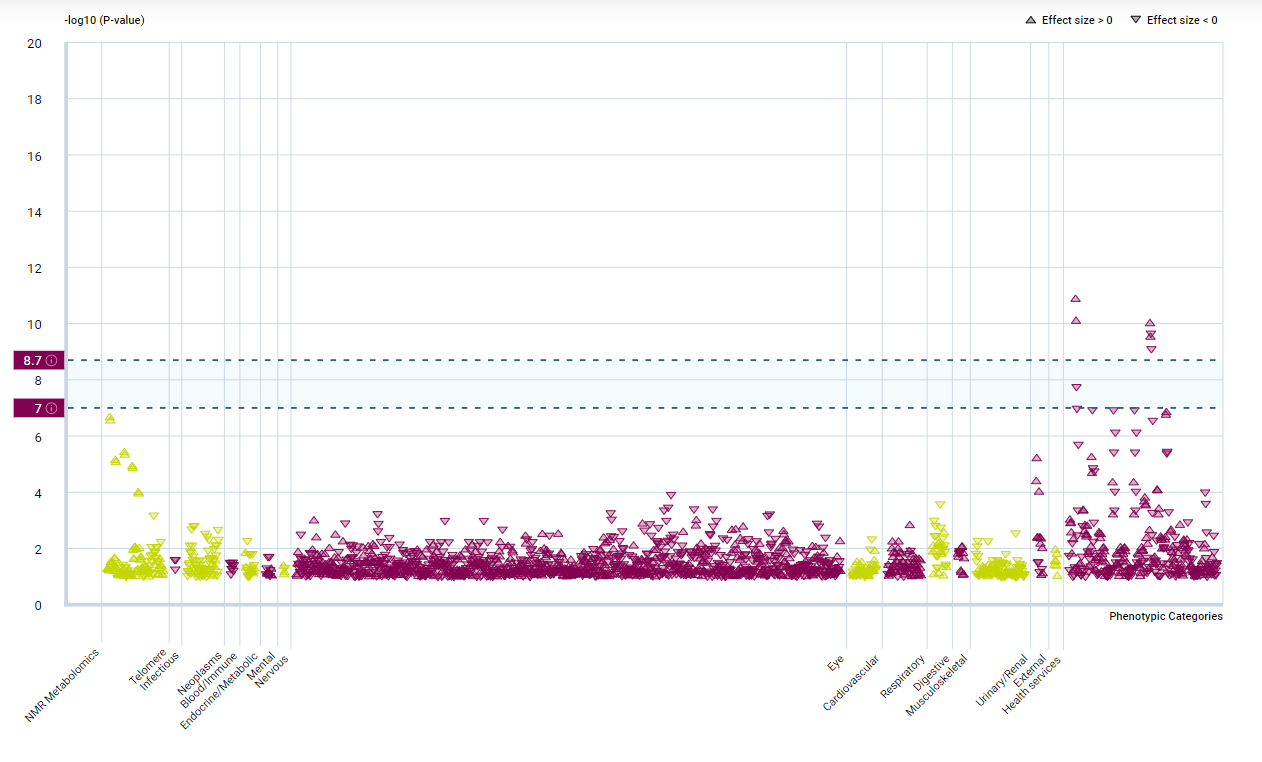


Figure S14. Continuous traits PheWAS association with ATP2A1
